# INX-315, a Selective CDK2 Inhibitor, Induces Cell Cycle Arrest and Senescence in Solid Tumors

**DOI:** 10.1158/2159-8290.CD-23-0954

**Published:** 2023-12-01

**Authors:** Catherine Dietrich, Alec Trub, Antonio Ahn, Michael Taylor, Krutika Ambani, Keefe T. Chan, Kun-Hui Lu, Christabella A. Mahendra, Catherine Blyth, Rhiannon Coulson, Susanne Ramm, April C. Watt, Sunil Kumar Matsa, John Bisi, Jay Strum, Patrick Roberts, Shom Goel

**Affiliations:** 1 Sir Peter MacCallum Department of Oncology, The University of Melbourne, Parkville, Victoria, Australia.; 2 Peter MacCallum Cancer Centre, Melbourne, Australia.; 3 Incyclix Bio, Durham, North Carolina.; 4 Ayurveda Molecular Modeling, Telangana, Hyderabad, India.

## Abstract

Cyclin-dependent kinase 2 (CDK2) is thought to play an important role in driving proliferation of certain cancers, including those harboring *CCNE1* amplification and breast cancers that have acquired resistance to CDK4/6 inhibitors (CDK4/6i). The precise impact of pharmacologic inhibition of CDK2 is not known due to the lack of selective CDK2 inhibitors. Here we describe INX-315, a novel and potent CDK2 inhibitor with high selectivity over other CDK family members. Using cell-based assays, patient-derived xenografts (PDX), and transgenic mouse models, we show that INX-315 (i) promotes retinoblastoma protein hypophosphorylation and therapy-induced senescence (TIS) in *CCNE1-*amplified tumors, leading to durable control of tumor growth; (ii) overcomes breast cancer resistance to CDK4/6i, restoring cell cycle control while reinstating the chromatin architecture of CDK4/6i-induced TIS; and (iii) delays the onset of CDK4/6i resistance in breast cancer by driving deeper suppression of E2F targets. Our results support the clinical development of selective CDK2 inhibitors.

**Significance:**

INX-315 is a novel, selective inhibitor of CDK2. Our preclinical studies demonstrate activity for INX-315 in both *CCNE1-*amplified cancers and CDK4/6i–resistant breast cancer. In each case, CDK2 inhibition induces cell cycle arrest and a phenotype resembling cellular senescence. Our data support the development of selective CDK2 inhibitors in clinical trials.

*See related commentary by Watts and Spencer, p. 386.*

*This article is featured in Selected Articles from This Issue, p. 384*

## Teaser

The CDK2 inhibitor INX-315 induces cell cycle arrest and therapy-induced senescence, thereby controlling the growth of *CCNE1*-amplified cancers and CDK4/6 inhibitor-resistant breast cancers, which together supports future clinical development.

## INTRODUCTION

Given that cancer is fundamentally a disease of uncontrolled cellular proliferation, the development of therapies that specifically target cell cycle progression has been a long sought-after goal for the cancer drug development community (1). However, many early attempts to develop inhibitors of CDK were unsuccessful due to poor compound selectivity resulting in unfavorable toxicity profiles (2). Indeed, the first FDA approval for a CDK inhibitor was not until 2015, when the selective CDK4/6i palbociclib entered clinical practice as treatment for hormone receptor (HR)-positive breast cancer (3). As a class, the approved CDK4/6i have subsequently provided proof of principle that the selective targeting of CDKs not only reduces drug toxicity but can also significantly improve survival outcomes (1).

CDKs 4 and 6 regulate a cell's passage from the G_1_ to the S-phase of the cell cycle by phosphorylating and hence inactivating the retinoblastoma (Rb) tumor suppressor protein, which in turn derepresses E2F-mediated transcription. By blocking Rb phosphorylation, CDK4/6i induce G_1_ arrest and a phenotype resembling senescence in sensitive cancer cells (4). CDK2 is the other CDK that primarily operates during the G_1_ and S cell cycle phases. Like CDK4/6, CDK2 directly phosphorylates Rb but also phosphorylates numerous other substrates required for the G_1_–S transition, the initiation of DNA replication, DNA repair, and eventual exit from S-phase (5). CDK2 is activated by binding either E- or A-type cyclins and by a range of protein kinases that directly phosphorylate CDK2. Conversely, it is inhibited by the endogenous CDK interacting protein/Kinase inhibitory proteins p21, p27, and p57 and the ubiquitination of its partner cyclins (5).

Like CDKs 4 and 6, there are cogent arguments supporting the development of selective CDK2 inhibitors for certain cancers. First, various genetic alterations can upregulate cyclin E1 protein levels and consequently increase CDK2 activity. Most notable of these is amplification of *CCNE1*, observed in a significant fraction of high-grade serous ovarian carcinomas (HGSOC), gastro-esophageal carcinomas, and uterine serous carcinomas (6). In these cancers, *CCNE1* amplification is associated with high levels of cyclin E1 protein, CDK2-dependent proliferation, chemotherapy resistance, and a poor prognosis (7–10). In addition, some lung, colorectal, and hematopoietic cancers inactivate the F-box family protein FBXW7, thereby inhibiting cyclin E1 degradation (11). Second, acquired resistance to CDK4/6 inhibition in luminal breast cancers may be driven by cyclin E/CDK2 activity (facilitating S-phase entry despite CDK4/6 blockade), providing rationale for studying the inhibition of CDK2 in these tumors as well (12–14). Despite these points, controversy about the therapeutic relevance of the CDK2 kinase as a target in cancer therapy persists for a variety of valid reasons (15), and the availability of a selective CDK2 inhibitor would help resolve a number of these outstanding questions.

Recently PF-06873600, a pharmacologic inhibitor targeting all three major G_1_–S CDKs (a CDK2/4/6 inhibitor), was described (14). The efficacy of this agent in preclinical models of both *CCNE1-*amplified and CDK4/6i–resistant cancers was encouraging and supported the rationale for targeting CDK2 in cancer, but its clinical development has been discontinued (16). The development of NUV-422, a second CDK2/4/6 inhibitor, was also discontinued on account of prohibitive toxicity (17). These experiences, coupled with a knowledge of a specific role for CDK2 in driving growth of certain cancers, has driven efforts to develop compounds that selectively inhibit CDK2.

There are numerous theoretical advantages to the use of selective CDK2 inhibitors rather than compounds that inhibit all G_1_–S CDKs. First, a selective CDK2 inhibitor would allow for the sole targeting of CDK2 in cancers where CDK2 is thought to be the primary driver (e.g., *CCNE1-*amplified tumors), avoiding the concomitant (and potentially dose-limiting) toxicity of inhibiting CDKs 4 and 6. Second, in cancers where combined CDK4/6 and CDK2 inhibition is warranted, separation of these components allows for flexibility with dosing, increasing the chances of finding an effective and tolerable treatment schedule. Third, selective CDK2 inhibitors could play an important role as preclinical tools to dissect the relative contributions of CDK2 and CDK4/6 in driving the proliferation of various cell types, and importantly the extent to which these kinases exhibit plasticity to sustain proliferation when one or the other is inhibited. For example, in many cell types, acute pharmacologic inhibition of CDK2 kinase activity may be rapidly compensated for by CDK4/6, sustaining Rb phosphorylation and S-phase entry (18). Whether this is true in *CCNE1*-amplified cancers, which might be highly CDK2-dependent (19), remains unclear. Similarly, if CDK2 drives proliferation of luminal breast cancers that have acquired resistance to CDK4/6 inhibition, it is not clear whether this reflects a change to a state of primary CDK2 dependence, or whether combined inhibition of CDK2 and CDK4/6 is needed to regain control of tumor cell proliferation.

A number of putative selective CDK2 inhibitors have now entered early-phase clinical development including INX-315 (Incyclix Bio), PF-07104091 (Pfizer), BLU-222 (Blueprint Medicine), INCB123667 (Incyte), and ARTS-021 (Allorion Therapeutics). To date, however, neither the selectivity profiles of these compounds nor their effects in preclinical models of cancer have been reported. Here, we report the discovery and development of INX-315, a selective and potent CDK2 inhibitor. We also describe the effects of INX-315 in preclinical models of *CCNE1-*amplified cancer and use the compound to dissect out the roles of CDK2 and CDK4/6 in treatment-naïve and therapy-resistant luminal breast cancers.

## RESULTS

### INX-315 Is a Potent and Selective CDK2 inhibitor

**Figure 1.**
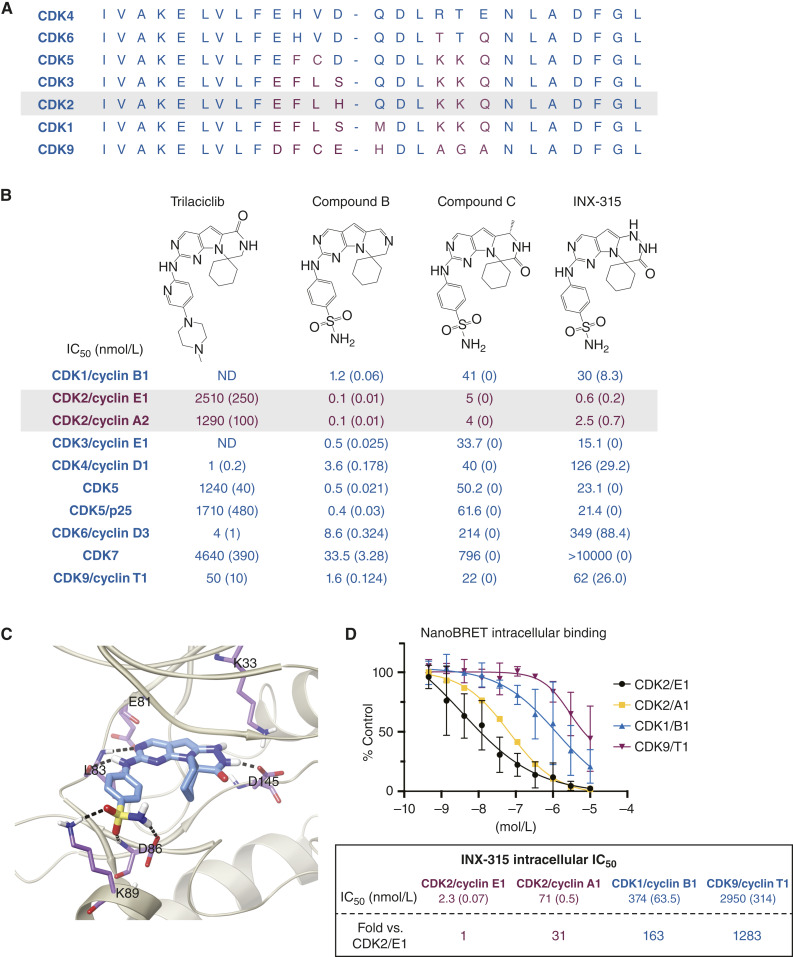
Discovery and characterization of INX-315. **A,** Amino acid sequence homology at the ATP-binding pockets of several CDKs. **B,** Derivation of INX-315 through serial modifications of trilaciclib, a CDK4/6i. Table indicates biochemical IC_50_s (± SEM) to cyclin/CDK pairings shown using the Nanosyn biochemical assay. Trilaciclib IC_50_ for CDK1/cyclin B1 was ND (for trilaciclib: *n* = 6 for CDK2/cyclin E1 and CDK2/cyclin A2, *n* = 9 for CDK4/cyclin D1, *n* = 3 for other complexes; for compound B: *n* = 3 for all complexes; for compound C: *n* = 1 for all complexes; for INX-315: if SEM is 0, *n* = 1, otherwise *n* = 6). **C,** Modeling of INX-315 bound to cyclin E1/CDK2. **D,** NanoBRET assay quantifying INX-315's intracellular displacement of tracer from the ATP-pocket of the cyclin/CDK pairings shown. Table shows calculated IC_50_s (two technical replicates per experiment, two biological replicates except for CDK2/Cyclin A1 where *n* = 1; error bars represent SD).BID, twice daily; QD, once daily.

Previous attempts to develop selective CDK2 inhibitors were unsuccessful due to poor selectivity and in particular due to unintended activity against CDKs 1, 4, 6, and/or 9 (2). The difficulty of developing selective CDK inhibitors is underpinned by the high degree of amino acid conservation in the ATP-binding pockets of these enzymes (Fig. 1A). In attempting to generate a highly potent and selective CDK2 inhibitor, we began with the FDA-approved selective CDK4/6i trilaciclib and modified its structure to reveal structure–activity relationships over numerous design cycles (20). This ultimately led to the derivation of INX-315, a highly potent and selective CDK2 inhibitor and clinical candidate (Fig. 1B; Supplementary Fig. S1A; Supplementary Synthetic Methods; Supplementary Table S1). The high level of selectivity of trilaciclib for CDK4/6 relative to CDK2 and CDK1 (20) was lost when the lactam carbonyl was removed and the methyl piperazine side chain was replaced with a benzyl sulfonamide group attached to the hinge nitrogen, generating compound B (Fig. 1B). The attachment of a benzyl sulfonamide group demonstrated significantly increased potency for CDKs 1, 2, and 9; however, the metabolic stability was poor (Fig. 1B; Supplementary Fig. S1B and S1C). Modifications on the tricyclic core generated compound C that showed decreased potency across all CDKs tested and poor metabolic stability (Fig. 1B; Supplementary Fig. S1B). To improve metabolic stability, a pyridazine modification in the tricyclic core of compound C generated INX-315, which greatly improved the metabolic stability across all species (Supplementary Fig. S1B). The addition of the carbonyl moiety in compound C proved to be critical to the chemical stability of INX-315 as the pyridazine modification without the carbonyl moiety in compound C yielded a compound that was chemically unstable. In addition, there was an approximate 10-fold increase in potency on CDK2 and improved selectivity over CDKs 1, 4, 6, and 9 relative to compound C (Fig. 1B; Supplementary Fig. S1D). While the IC_50_ for CDK1 decreased slightly, the selectivity of INX-315 for CDK2 compared with CDK1 increased to 50-fold. This surprising observation will be the subject of further studies to understand which residues are providing for the enhanced selectivity. As observed in the modeling of INX-315 bound to CDK2/cyclin E1 (Fig. 1C), there are multiple interactions of D86 and K89 with the sulfonamide moiety and an additional interaction with D145. The core interactions with the hinge backbone residue L83 remain strong and unaltered and correlate well with the high binding affinity toward CDK2 observed in biochemical assays.

To further characterize INX-315, we used NanoBRET live cell target engagement assays to assess the intracellular selectivity of INX-315. The intracellular target engagement IC_50_ for INX-315 and CDK2/cyclin E1 complexes was 2.3 nmol/L, only a 4-fold increase over the biochemical IC_50_ (Fig. 1D). The intracellular IC_50_ for INX-315 and CDK1/cyclin B1 was 374 nmol/L while the IC_50_ for CDK9/cyclin T1 was 2,950 nmol/L (Fig. 1D). Thus, INX-315 shows even greater selectivity for CDK2 in the intracellular NanoBRET assay than in biochemical assays.

While several companies have begun clinical trials with putative CDK2 inhibitors, only Pfizer's selective CDK2 inhibitor (PF-07104091) has a published structure, enabling us to compare its potency, selectivity, and efficacy with INX-315. PF-07104091's biochemical IC_50_ for CDK2/cyclin E1 was 2.4 nmol/L, 4-fold greater than INX-315. While PF-07104091 has greater selectivity over CDKs 4, 6, and 9, INX-315 is more selective against CDK1 (Supplementary Fig. S1C). Looking at intracellular IC_50_s, INX-315 showed greater potency against CDK2/cyclin E1 in the NanoBRET assay (2.3 vs. 32 nmol/L) and a greater selectivity over CDK1 and 9 compared with PF-07104091 (Supplementary Fig. S1C and S1E).

We next assessed the broader kinase selectivity profile of INX-315 using the LanthaScreen Eu Kinase Binding and Z’-Lyte Kinase Assays. Treatment with 100 nmol/L INX-315 caused ≥94% inhibition of CDK2/cyclin A/A1/E1/O and caused ≥80% inhibition of CDK3/cyclin E1, CDK5 (inactive), CDK5/p25, CDK5/p35, colony stimulating factor 1 receptor (CSF1R), MAPK15/ERK7, neurotrophic tyrosine receptor kinase (NTRK)/tyrosine receptor kinase (TRK)C, and tyrosine receptor kinase (TYK)2 (Supplementary Table S2). These kinases were further assessed as dose–response curves from 50 pmol/L to 1 µmol/L. Follow-up experiments showed INX-315 displayed selectivity for CDK2/cyclin A1/E1/O with IC_50_ values of 4 nmol/L or less. CSF1R had an IC_50_ of 2.29 nmol/L, while all other targets had IC_50_ values greater than 10 nmol/L (Supplementary Fig. S1F). Taken together, these findings demonstrate selectivity of INX-315 for CDK2/cyclin A1/E1/O over most of the other kinases tested. These findings also identify the inhibition of CSF1R (a receptor for colony-stimulating factor 1 that mediates macrophage production and function) CDK3, and CDK5 as having the potential for off-target activity of INX-315.

### CDK2 Inhibition Results in Cell Cycle Arrest, a Senescence-Like State, and Tumor Growth Inhibition in *CCNE1-*amplifed Cancers

**Figure 2.**
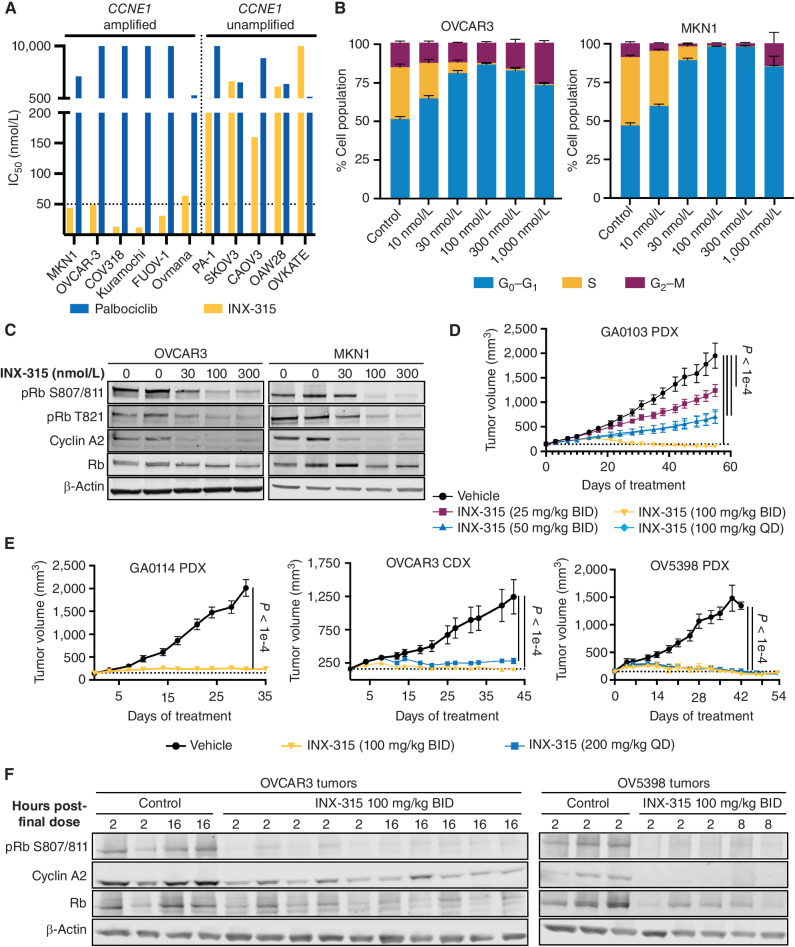
Activity of INX-315 in *CCNE1-*amplified cancers. **A,** IC_50_s for palbociclib and INX-315 for cell lines shown using CellTiter Glo viability assay. All cells were treated for 6 days, *n* = 3 technical replicates per cell line. **B,** Cell cycle phase profiles of OVCAR-3 and MKN1 cells treated with INX-315. All cells were treated for 24 hours, *n* = 3 technical replicates per cell line; error bars are SD. **C,** Western blots for phosphorylated/total Rb and cyclin A2 in OVCAR3 and MKN1 cells treated with INX-315 for 24 hours. **D** and **E,** Tumor growth curves for *CCNE1-*amplified gastric and ovarian carcinoma models treated with INX-315 at doses shown (GA0103 *n* = 8 per group; GA0114 *n* = 8 per group; OVCAR3 *n* = 10 per group; OV5398 *n* = 10 per group; error bars represent SEM; *P* values calculated using two-way ANOVA. all experiments performed once). **F,** Western blots for phosphorylated/total Rb and cyclin A2 in tumor tissue lysates treated with control vehicle or INX-315. Samples were collected at experimental endpoint from experiments in **E**.

We first set out to determine the impact of INX-315 treatment in preclinical models of *CCNE1-*amplified cancers. We treated a panel of ten ovarian cancer cell lines (including five HGSOC with *CCNE1* amplification) with either INX-315 or palbociclib for six days, allowing for multiple doubling times before assessment of cell viability. INX-315 had a cellular IC_50_ of < 100 nmol/L (mean 36 nmol/L, range 10–64 nmol/L) in the five cell lines with *CCNE1* amplification (copy number > 2), but a mean IC_50_ of 1,435 nmol/L (range 159–3560 nmol/L) in the five lines without *CCNE1* amplification (Fig. 2A; Supplementary Fig S2A; ref. 21). *CCNE1-*amplified lines were not sensitive to palbociclib, suggesting that their proliferation over this time frame is primarily CDK2-dependent, but not CDK4/6-dependent (Fig. 2A). *CCNE1* amplification also occurs commonly in gastric carcinoma, so we tested the cell line MKN1 and found it was similarly sensitive to INX-315 (IC_50_ 44 nmol/L) and insensitive to palbociclib (Fig. 2A). When compared with PF-07104091, INX-315 had a lower IC_50_ for all *CCNE1-*amplified lines (mean 36 nmol/L vs. 125 nmol/L), consistent with INX-315 having a lower biochemical and intracellular IC_50_ for CDK2/cyclin E1 (Supplementary Fig. S2B). In addition, we observed that the benign human fibroblast cell line Hs68 was sensitive to both palbociclib (IC_50_ 26 nmol/L) and the pan-CDK inhibitor dinaciclib (IC_50_ 7 nmol/L) but not INX-315 (Supplementary Fig. S2C). This suggests that unlike in normal cells where CDK4/6 can rapidly compensate for pharmacologic inhibition of CDK2 to drive ongoing proliferation (18), this is not the case in *CCNE1*-amplified cancers.

As INX-315 inhibits CDK2/cyclin E1 (regulating G_1_ to S transition) more potently than CDK2/cyclin A2 (regulating transit through and exit from S-phase), we predicted that sensitive cells would primarily arrest in G_1_ following INX-315 treatment. In OVCAR3 cells, we observed a potent G_1_ arrest after 24 hours of treatment with as little as 30–100 nmol/L INX-315 (Fig. 2B; Supplementary Fig. S3A). Similarly, MKN1 cells showed a strong G_1_ arrest at 100 nmol/L following INX-315 treatment (Fig. 2B; Supplementary Fig. S3A). At higher concentrations, we observed a shift from a G_1_ arrest to a combined G_1_/G_2_ arrest (Fig. 2B; Supplementary Fig. S3A), potentially reflecting increased inhibition of CDK2/cyclin A2 or CDK1/cyclin B1 at these doses. Identifying the mechanism and consequences of this shift merits further investigation. The G_1_ cell cycle arrest was accompanied by a reduction in levels of phosphorylated Rb, CDC6, and nucleolin consistent with INX-315's action as a CDK2 inhibitor (Fig. 2C; Supplementary Fig. S4A and S4B). The effect on Rb phosphorylation started at doses as low as 30 nmol/L and increased up to 300 nmol/L (Fig. 2C). In addition, we observed a marked reduction in the protein level of the E2F target cyclin A2 (Fig. 2C). Compared with PF-07104091, INX-315 induced a stronger reduction in phosphorylation of CDK2 substrates at equivalent doses, demonstrating the greater potency of INX-315 against CDK2 (Supplementary Fig. S4A and S4B). In the non-amplified and insensitive cell line SKOV3, no sustained changes to CDK2 targets were observed during treatment with either CDK2 inhibitor (Supplementary Fig. S4C)

We next tested the *in vivo* efficacy of INX-315 using several mouse models of *CCNE1-*amplified cancer. We first used the gastric adenocarcinoma PDX GA0103 (<10 copies *CCNE1* per cell; ref. 22). Mice were treated with 25, 50, or 100 mg/kg of INX-315 (oral gavage) twice daily for 56 days. An additional experimental group was dosed with 100 mg/kg once a day. Tumor growth was inhibited in a dose-dependent manner with the highest dose (100 mg/kg twice daily) group showing tumor regression and significant growth inhibition observed in all other treatment groups (Fig. 2D). We selected the 100 mg/kg twice daily dose and tested three additional *in vivo* models of *CCNE1-*amplified cancer: a second PDX model of gastric adenocarcinoma (GA0114, *CCNE1* copy number <3), an ovarian carcinoma PDX model OV5398 (*CCNE1* copy number <9), and a cell-derived xenograft (CDX) model OVCAR-3 (*CCNE1* copy number <10; ref. 22). The growth of GA0114 tumors was significantly inhibited for the duration of the 5-week experiment (Fig. 2E). Similar efficacy was observed in the OVCAR-3 and OV5398 models, with the latter experiment running for 8 weeks. No mice lost more than 5% of their body weight during the studies and mice treated with INX-315 did not display any concerning features suggestive of drug toxicity such changes in body condition score, respiratory rate, coat condition, posture, or behavior (Supplementary Fig. S4D). Both ovarian carcinoma models showed reductions in Rb phosphorylation and cyclin A2 in tumor tissue at the experimental endpoint (between 44 and 54 days of treatment), sustained up to 16 hours after the final dose was administered (Fig. 2F). This confirms that prolonged CDK2 inhibition *in vivo* can exert sustained control of *CCNE1-*amplified tumors, and that rebound compensation by CDK4/6 is not present to a level sufficient to significantly restore Rb phosphorylation, cyclin A2 expression, or tumor growth.

**Figure 3.**
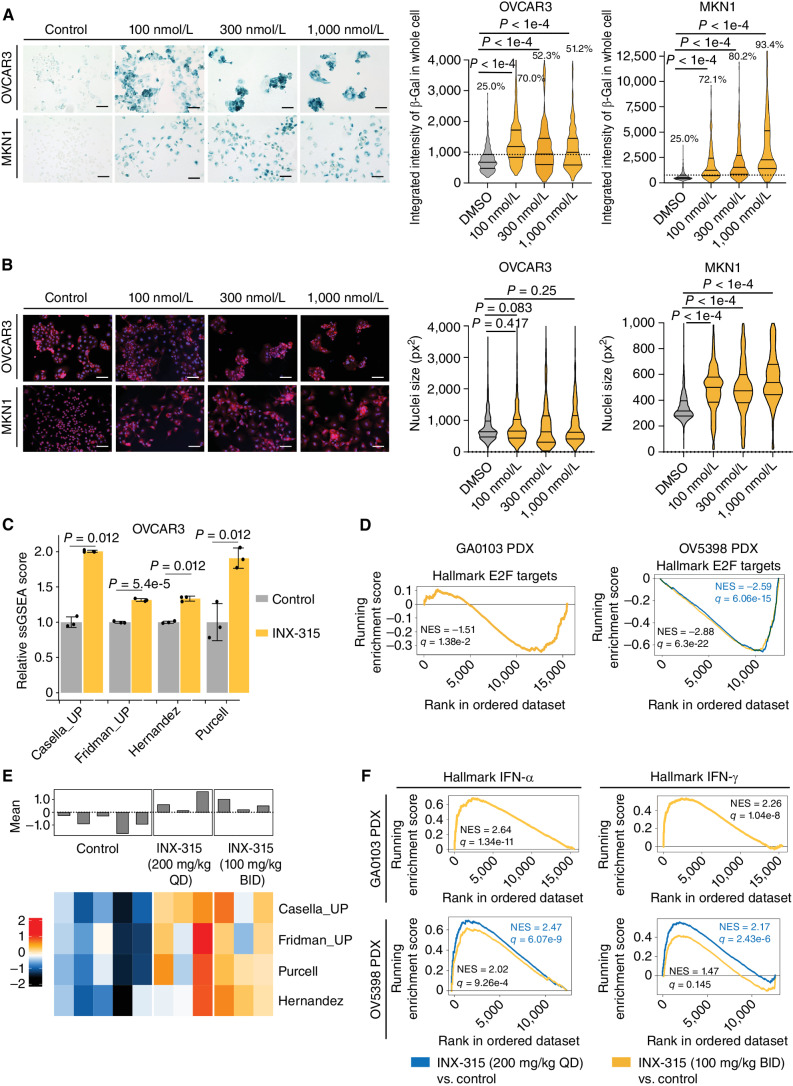
TIS after INX-315 treatment of *CCNE1-*amplified cancers. **A,** Left, representative images after staining OVCAR3 and MKN1 cells for beta-galactosidase activity after treatment with INX-315 (scale bar = 100 µm). Experiments performed with three technical replicates. Right, quantification of integrated beta-galactosidase signal per cell. Dashed line indicates cutoff used to define beta-galactosidase positivity, and numbers represent percentage of beta-galactosidase–positive cells. **B,** Left, representative images after staining OVCAR3 and MKN1 cells with DAPI and phalloidin after treatment with INX-315 (scale bar = 100 µm). Right, quantification of relative nuclear size based on DAPI staining. Experiments performed with three technical replicates. **C,** ssGSEA scores derived from RNA sequencing of OVCAR3 cells treatment with control or INX-315 300 nmol/L for seven days. Scores are calculated for four independent senescence-associated gene sets; three technical replicates. **D,** GSEA plots derived from RNA-sequencing performed on tumor tissue from experiments in Fig. 2D and E. Normalized enrichment score (NES) and q value (false discovery rate) were calculated as described in Methods (all *P* values were calculated using unpaired *t* tests, error bars represent SD). **E,** ssGSEA scores for four senescence-associated gene signatures were calculated from RNA-sequencing on OV5398 PDX tumor tissue. Heat map shows z-scores for these. Bar plot above shows mean z-score for each sample across all signatures. **F,** GSEA plots derived from RNA-sequencing performed on tumor tissue from experiments in Fig. 2D and E. NES and q value (false discovery rate) were calculated as described in Methods (all *P* values were calculated using unpaired *t* tests, error bars represent SD).

In luminal breast cancer, hypophosphorylation and hence activation of Rb by CDK4/6i induces a tumor cell phenotype resembling cellular senescence (4, 23, 24). In that context, the induction of therapy-induced senescence (TIS) is linked to the activation of a tumor cell–intrinsic IFN response and enhanced tumor cell immunogenicity (25). Moreover, these agents improve overall survival of patients with breast cancer suggesting that induction of TIS by cell cycle inhibitors is a promising strategy for the treatment of solid tumors (26, 27). Given these observations, we next sought to determine whether inhibition of Rb phosphorylation through selective CDK2 inhibition would also induce a TIS phenotype in *CCNE1-*amplified cancers. To this end, we treated OVCAR3 and MKN1 cells with INX-315 (100 nmol/L, 300 nmol/L, 1,000 nmol/L) for 7 days and measured cellular β-galactosidase activity as a marker of TIS. INX-315 significantly increased tumor cell β-galactosidase activity in both cell lines at all doses tested (Fig. 3A). In MKN1 cells, this was accompanied by a significant increase in cell size and also nuclear size, another hallmark of senescence (Fig. 3B). RNA-sequencing of OVCAR3 cells treated with INX-315 for 7 days provided orthogonal evidence for INX-315–induced TIS in these cells: (i) expression of E2F target genes was markedly reduced (Supplementary Fig. S5A); (ii) the expression of genes within four independent and nonoverlapping gene sets linked to cellular senescence (28) was consistently upregulated (Fig. 3C); (iii) the expression of genes encoding AP-1 transcription factors, orchestrators of the senescence program in a variety of contexts (29, 30), was upregulated (Supplementary Fig. S5B).

We next performed RNA-sequencing on tumor tissue collected at experimental endpoint (<40–50 days) after mice bearing OV5398 (ovarian carcinoma) and GA0103 (gastric carcinoma) PDX tumors had been treated with control vehicle or INX-315. Consistent with our *in vitro* data, both PDX models showed suppression of E2F target gene expression, upregulation of genes within multiple senescence gene signatures, upregulation of *CDKN1A* and *BCL2L1* expression (both often increased in senescent cells), and significant upregulation of genes indicating a response to IFNs (Fig. 3D–F; Supplementary Fig. S5C). These findings support the notion that prolonged CDK2 inhibition *in vivo* can not only arrest the proliferation of *CCNE1*-amplified cancer cells, but also induce a TIS phenotype.

### CDK2 Inhibition Restores Sensitivity to CDK4/6 Inhibition in Luminal Breast Cancer

Although combined antiestrogen therapy (ET) plus CDK4/6 inhibition is a highly effective therapy for HR-positive breast cancer, therapeutic resistance remains a common and poorly understood problem (1). Preclinical studies suggest that although proliferation of these tumors is primarily CDK4/6-dependent, resistance to CDK4/6i monotherapy might be driven by CDK2 activity (13, 19), and clinical data has shown a correlation between high tumor *CCNE1* mRNA levels and impaired response to combined CDK4/6i +/- ET (12, 31). These data provide a rationale to determine the role of CDK2 inhibition in CDK4/6i–resistant tumors.

To this end, we generated cell line models of therapy-resistant luminal breast cancer by culturing MCF7 (p53 wild-type) and T47D (p53-mutant) cells in increasing concentrations of the CDK4/6i abemaciclib, the selective estrogen receptor degrader fulvestrant, or their combination until resistance was observed (defined as sustained growth in 500 nmol/L of abemaciclib, 100 nmol/L of fulvestrant, or 500 nmol/L/100 nmol/L, respectively, for the combination; Supplementary Fig. S6A). As expected, short-term abemaciclib treatment induced potent suppression of E2F target genes in both parental cell lines, which was restored in abemaciclib- and abemaciclib/fulvestrant–resistant cells growing in drug (Supplementary Fig. S6B).

**Figure 4.**
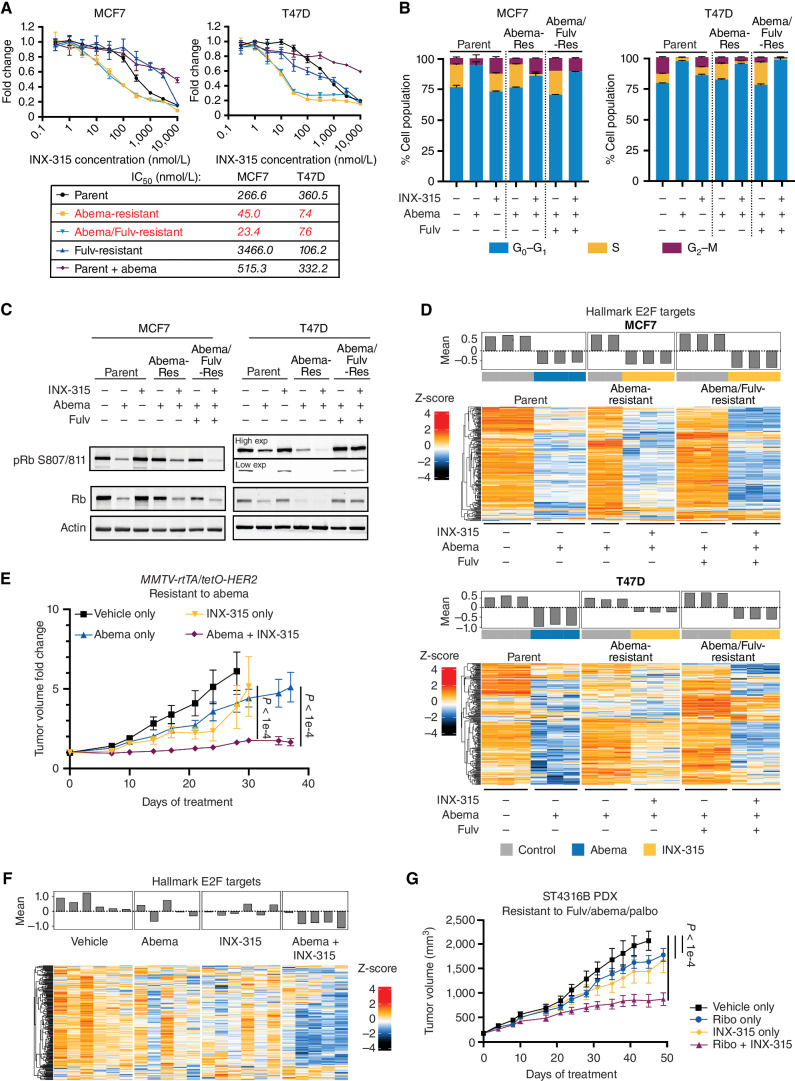
Activity of INX-315 in CDK4/6i–resistant breast cancer. **A,** Dose–response curves for MCF7 and T47D cell lines treated with INX-315 for 7 days. Parent, parent cells in DMSO; Abema-resistant, abemaciclib-resistant cells growing in 500 nmol/L abemaciclib; Abema/Fulv-resistant, resistant to abemaciclib/fulvestrant growing in 500 nmol/L abemaciclib plus 100 nmol/L fulvestrant; Fulv-resistant, fulvestrant resistant growing in 100 nmol/L fulvestrant; Parent + abema, parental cells treated with 500 nmol/L abemaciclib and INX-315 concurrently. Table shows IC_50_s for INX-315 in each case, derived from measurement of cell number (error bars represent SD; two biological replicates, six technical replicates each time). **B,** Cell cycle phase profiles of MCF7 and T47D cells (and parental and drug-resistant) treated with drugs shown for 7 days (500 nmol/L abemaciclib; 100 nmol/L fulvestrant; INX-315, 300 nmol/L for MCF7, 100 nmol/L for T47D; values are mean of experiments performed in duplicate, error bars are SD). **C,** Western blots for phosphorylated/total Rb in MCF7 and T47D cells treated as in **B**. **D,** Heat map showing z scores for individual E2F target genes (RNA-sequencing) in MCF7 and T47D cells treated as in **B**. Bar plot above shows mean z-score for all genes in each sample. Three technical replicates per condition. **E,** Tumor growth curves for *MMTV-rtTA/tetO-HER2* tumors treated with control vehicle (*n* = 17 tumors in 6 mice), abemaciclib (*n* = 17 tumors in 6 mice), INX-315 (*n* = 20 tumors in 6 mice), or the combination (*n* = 15 tumors in 6 mice). Tumors were pretreated with abemaciclib for 3–4 weeks prior to randomization, at which point abemaciclib resistance was present. Experiment was repeated twice (error bars represent SEM; *P* values calculated using two-way ANOVA). **F,** Heat map showing z scores for individual E2F target genes (RNA-sequencing) in *MMTV-rtTA/tetO-HER2* tumors from **E**. Bar plot above shows mean z-score for all genes in each sample. Six samples for vehicle and INX-315, five samples for abemaciclib and combination. **G,** Tumor growth curves for ST4316B PDX tumors treated with control, ribociclib, INX-315, or the combination (*n* = 8 per group; error bars represent SEM; *P* values calculated using two-way ANOVA).

Both abemaciclib- and abemaciclib/fulvestrant–resistant cells expressed higher levels of *CCNE1* mRNA and protein than their parental counterparts, consistent with the notion that heightened CDK2 activity might drive their proliferation in the face of sustained CDK4/6 inhibition (Supplementary Fig. S7A and S7B). This notion was further supported by the fact that knockdown of *CCNE1* with siRNA induced near complete cell cycle arrest in abemaciclib- and abemaciclib/fulvestrant–resistant MCF7 cells (Supplementary Fig. S7C). Importantly, when cultured continuously in drug(s) to which they were resistant, cells that had acquired resistance to CDK4/6 inhibition (either as a single agent or in combination with fulvestrant) were markedly more sensitive to INX-315 than (i) untreated parental cells; (ii) fulvestrant-resistant cells; or (iii) parental cells treated upfront with concomitant abemaciclib (Fig. 4A). These experiments were conducted by treating cells with INX-315 for two doubling times, thus accounting for the variable proliferation rates of different drug-resistant cells (32). Similar data were obtained with PF-07104091, although again IC_50_ values were higher than for INX-315 (Supplementary Fig. S7D–S7F). These data demonstrate not only that the proliferation of parental cells is heavily CDK4/6 (rather than CDK2)-driven, but also that the state of acquired CDK4/6i resistance (both to monotherapy and to combinations with endocrine therapy) is associated with heightened sensitivity to CDK2 inhibition.

To explore the relative contributions of proliferative arrest and cell death underlying the activity of INX-315 in CDK4/6i–resistant breast cancer, we treated parental cells with abemaciclib or INX-315 and treated resistant cells (cultured in drug(s) to which they were resistant) with INX-315 prior to measuring indices of proliferation and apoptosis. Although abemaciclib induced complete cell cycle arrest in parental cells (<1% of cells in S-phase), INX-315 only reduced the S-phase fraction modestly (Fig. 4B; Supplementary Fig. S8A). In contrast, once cells had acquired resistance to CDK4/6i therapy, their proliferation was potently suppressed by INX-315, evidenced by near complete loss of cells in S-phase and an increase in the proportion of cells in G_1_ (Fig. 4B; Supplementary Fig. S8A). This proliferative arrest was accompanied by a reduction in Rb phosphorylation and also a reduction in phosphorylation of nucleolin (Fig 4C; Supplementary Fig. S8B). In contrast, cleavage of PARP – an index of apoptosis – was unchanged or reduced upon treatment of CDK4/6i–resistant cells with INX-315 (Supplementary Fig. S8C).

To determine the impact of selective CDK2 inhibition with INX-315 on the transcriptome of CDK4/6i–resistant breast cancer, we treated abemaciclib and abemaciclib/fulvestrant–resistant cells with INX-315 and performed RNA-sequencing. Consistent with earlier results, expression of cell cycle–related genes (including E2F targets) was heavily suppressed by abemaciclib treatment of parental cells, restored in drug-resistant cells, and suppressed again when INX-315 was added to resistant cells (Fig. 4D). Collectively, these data demonstrate that acquired resistance to CDK4/6i therapy (including in combinations with endocrine therapy) is associated with CDK2 dependency that can be targeted by INX-315 leading to restored control of proliferation at the G_1_–S boundary.

We next sought to determine whether the sensitivity of CDK4/6i–resistant cells to INX-315 reflects a switch in the dependence of these cells from CDK4/6 to CDK2 (i.e., that a CDK2 inhibitor alone could induce their cell cycle arrest), or if in fact maximal control of resistant cell proliferation required coinhibition of CDK2 and CDK4/6. The growth of abemaciclib-resistant MCF7 and T47D cells accelerated when they were cultured out of abemaciclib, suggesting that the CDK4/6i was still exerting some antiproliferative effect in the setting of resistance (Supplementary Fig. S9A). Furthermore, although INX-315 monotherapy slowed the growth of these cells, the combination of INX-315 and abemaciclib reduced viability significantly more than either agent alone. This was true with or without the concomitant use of fulvestrant (Supplementary Fig. S9A). Collectively this suggests that although increased CDK2 activity is sufficient to drive proliferation (i.e., resistance) in the presence of sustained CDK4/6 inhibition, optimal growth control of these resistant cells requires CDK2 inhibition plus sustained inhibition of CDK4/6. We observed similar results with MCF7 cells cultured to resistance in palbociclib, a different CDK4/6i (Supplementary Fig. S9B–S9E). When palbociclib-resistant MCF7 cells were treated with a variety of CDK4/6i as monotherapy (palbociclib, abemaciclib, or lerociclib), they continued to proliferate. When cultured out of CDK4/6i, these cells were relatively insensitive to INX-315 (IC_50_ <900 nmol/L). However, if any of the CDK4/6i was added to the culture medium, the IC_50_ to INX-315 dropped markedly (14 nmol/L with palbociclib; 23 nmol/L with lerociclib; 28 nmol/L with abemaciclib; Supplementary Fig. S9B–S9D).

Studying the impact of novel therapies on tumors that have acquired resistance to CDK4/6i *in vivo* is challenging, as there are few if any animal models mimicking the most common clinical scenario – namely, a period of response to CDK4/6 inhibition followed by eventual tumor outgrowth. To this end, we next set out to develop and characterize a novel *in vivo* model of CDK4/6i resistance. We utilized *MMTV-rtTA/tetO-HER2* transgenic mice, which develop spontaneously arising, cyclin D1 and Rb-expressing, CDK4/6i–sensitive adenocarcinomas within developmentally normal, mature mammary glands (25, 33). Tumor regression (>25% reduction in tumor volume) was seen in 163 of 183 (89%) of tumors treated with abemaciclib monotherapy, simulating the regressions seen with CDK4/6i monotherapy in patients (Supplementary Fig. S10A; refs. 34, 35). By day 35 of treatment, 142 of 163 regressing tumors (87%) had resumed growth and sustained this for at least ten days, which we defined as “acquired resistance” (Supplementary Fig. S10A). Resistant tumors retained Rb expression (Supplementary Fig. S10B) and whole-exome sequencing of three resistant tumors revealed wild-type sequence and diploid copy number for genes potentially implicated in resistance including *Rb1*, *Rbl1*, *Rbl2*, *Ccne1*, *Ccne2*, *Cdk4*, *Cdk6*, *Pik3ca*, *Akt1*, *Kras*, and *Fgfr1–*4. As expected, tumors responding to short-term abemaciclib therapy showed reductions in tumor cell proliferation (reduced Ki67 and E2F target gene expression) and consistent with our *in vitro* data, resistant tumors showed restoration of these parameters to an intermediate level between that of untreated and responding tumors (Supplementary Fig. S10C and S10D).

To determine the contribution of CDK2 in driving proliferation of CDK4/6i–resistant *MMTV-rtTA/tetO-HER2* tumors, we treated cohorts of tumor-bearing mice with abemaciclib until acquired resistance was observed, and then randomly assigned mice to treatment with vehicle control, continued abemaciclib, INX-315, or their combination. Vehicle treatment (i.e., withdrawal of abemaciclib) led to acceleration of tumor growth accompanied by increased tumor cell proliferation and Rb phosphorylation, consistent with the notion that CDK4/6i continue to play some antiproliferative role in these tumors, even in the setting of resistance (Fig. 4E; Supplementary Fig. S10E–S10G). Although both continued abemaciclib monotherapy and INX-315 monotherapy slowed the growth of abemaciclib-resistant tumors compared with vehicle control, only their combination significantly slowed tumor growth over 5 weeks of treatment (Fig. 4E). RNA sequencing of tumor tissue mirrored these findings, with E2F target gene expression most heavily downregulated with combination treatment and most significantly upregulated with vehicle treatment (Fig. 4F). These data further suggest that the proliferation of Rb-proficient mammary tumors that have acquired CDK4/6i resistance is dependent upon both CDK4/6 and CDK2, and that optimal control of tumor growth requires their coinhibition.

We observed similar results in a PDX model (ST4316B) of CDK4/6i–resistant breast cancer. These tumors were derived from a patient with HR-positive breast cancer whose tumor demonstrated progression after treatment with fulvestrant/abemaciclib (1 month treatment) and also fulvestrant/palbociclib (3-month treatment; ref. 36). Like our transgenic model of CDK4/6i resistance derived *in situ*, treatment of this PDX with the CDK4/6i ribociclib or with INX-315 slowed tumor growth modestly, but their combination was significantly more effective (Fig. 4G). While all treatment groups initially lost weight, all recovered equally by the end of the study indicating the combination was well tolerated (Supplementary Fig. S10H).

### CDK2 Inhibition Reinstates Therapy-Induced Senescence in CDK4/6i–Resistant Breast Cancer

CDK4/6i therapy of treatment-naïve luminal breast cancers induces an Rb-dependent phenotype resembling cellular senescence (4). CDK4/6i–induced senescence is characterized by significant remodeling of the chromatin landscape, most notably the activation of genomic enhancers that drive transcriptional programs including luminal differentiation, the senescence-associated secretory phenotype (SASP), apoptotic evasion, and tumor cell immunogenicity–all phenotypic features of tumor cells responding to CDK4/6 inhibition (25, 30). We sought to determine whether reinduction of G_1_ arrest by INX-315 is accompanied by a reinstatement of a similar senescent phenotype.

**Figure 5.**
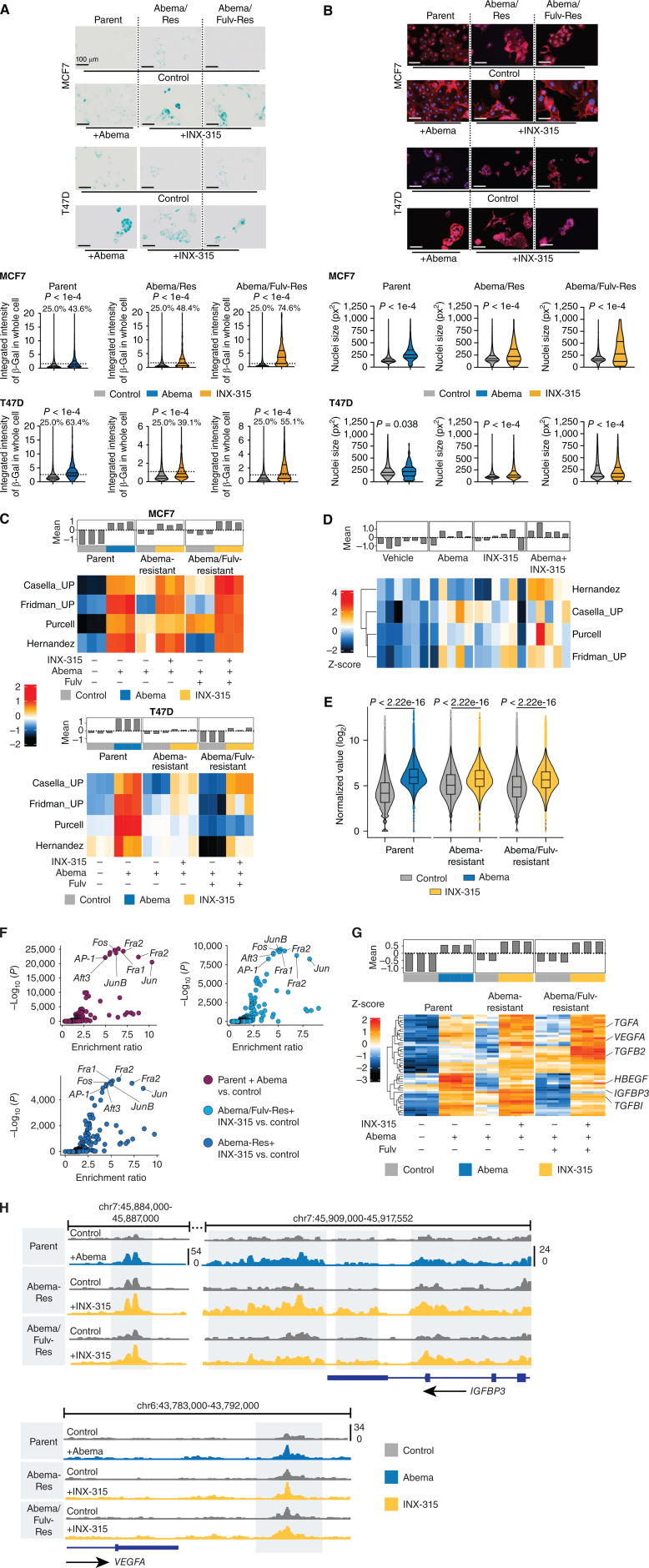
INX-315 treatment of CDK4/6i–resistant breast cancer reinstates a senescence phenotype. **A,** Representative images (top) and quantification (bottom) of staining MCF7 and T47D cells for beta-galactosidase activity after treatment with agents shown (500 nmol/L abemaciclib, INX-315: 300 nmol/L for MCF7 and 100 nmol/L for T47D) for 7 days (scale bar = 100 µm). Resistant cells were cultured continuously in drugs to which they were resistant (500 nmol/L abemaciclib ± 100 nmol/L fulvestrant). Experiments performed with three technical replicates. Quantification is for integrated beta-galactosidase signal per cell. Dashed line indicates cutoff used to define beta-galactosidase positivity. **B,** Representative images (top) and quantification of nuclear size (bottom) upon staining MCF7 and T47D cells with DAPI and phalloidin after treatment as in **A** (scale bar = 100 µm). Experiments performed with three technical replicates. **C,** Heat map shows z-scores for ssGSEA scores for four senescence-associated gene signatures, calculated from RNA-sequencing of MCF7 and T47D cells treated as in **A**. Bar plot above shows mean z-score for each sample across all signatures. Three technical replicates for all conditions except abemaciclib-resistant MCF7 in abemaciclib, which had two technical replicates. **D,** Heat map as in **C**, but for RNA-sequencing from abemaciclib-resistant *MMTV-rtTA/tetO-HER2* tumors treated as in Fig. 4E. Six samples for vehicle and INX-315, five samples for abemaciclib and combination. **E,** Violin plots showing log_2_-transformed normalized ATAC-seq counts for genomic regions that significantly increased chromatin accessibility after abemaciclib treatment of parental cells. Cells treated as in **A**, two technical replicates. **F,** Scatter plots showing significant enrichment of AP-1 motifs in the ATAC-seq up peaks after treatment with drugs as in **A**. **G,** Heat map showing z-scores for individual SASP genes (RNA-sequencing) in MCF7 cells treated as in **A**. Bar plot above shows mean z-score for all genes in each sample. Three technical replicates for all conditions except abemaciclib-resistant MCF7 in abemaciclib, which had two technical replicates. **H,** Representative ATAC-seq tracks at regions near SASP genes (*IGFBP3* and *VEGFA*) in cells treated as in **A** (all *P* values calculated using unpaired *t* tests except **F**, determined using HOMER package).

As expected, abemaciclib treatment of parental MCF7 and T47D cells induced changes consistent with cellular senescence (increased nuclear size and increased senescence-associated β-galactosidase activity), which were diminished at the time of acquired abemaciclib or abemaciclib/fulvestrant resistance. When resistant cells were treated with CDK4/6i and INX-315 for seven days, these parameters increased significantly, consistent with reinstatement of therapy-induced senescence (Fig. 5A and B). These observations were supported by analysis of RNA-sequencing data, which showed that INX-315 treatment of resistant cells upregulated genes within multiple senescence gene sets and altered the expression of established senescence-associated genes (e.g., upregulation of *CDKN1A*; Fig. 5C; Supplementary Fig. S11A). Notably, the same was true *in vivo*, with senescence-associated gene sets showing upregulation after combined abemaciclib and INX-315 treatment of abemaciclib-resistant *MMTV-rtTA/tetO-HER2* tumors (Figs. 4E and 5D).

We next sought to determine the extent to which the senescence phenotype induced by INX-315 treatment of CDK4/6i–resistant tumors was similar to, or distinct from, CDK4/6i–induced senescence in non-resistant tumor cells. Given the critical role of chromatin remodeling in governing the senescence phenotype (29, 30, 37), we performed the Assay for Transposase-Accessible Chromatin with Sequencing (ATAC-seq) on (i) parental cells treated with control vehicle or abemaciclib; (ii) abemaciclib- and abemaciclib/fulvestrant-resistant cells treated with control vehicle or INX-315. Chromatin regions that became less accessible after abemaciclib treatment of parental cells also showed reduced accessibility after INX-315 treatment of resistant cells and were heavily enriched for E2F target gene promoters or nonpromoter regions proximal to those genes (Supplementary Fig. S12A–S12C). Notably, the same was true for regions that showed a significant increase in accessibility after abemaciclib treatment, which were primarily situated over introns and distal intergenic regions (i.e., consistent with activated enhancers). When compared with abemaciclib-treated parental cells, resistant cells showed reductions in accessibility of these regions (consistent with partial reversion to a pre-senescent chromatin landscape), and INX-315 treatment of resistant cells led to “reopening” of these regions, consistent with reinstatement of a similar chromatin accessibility profile (Fig. 5E; Supplementary Fig. S12D and S12E).

In CDK4/6i–treated luminal breast cancers that have entered senescence, newly activated enhancers are heavily enriched for AP-1 transcription factor binding (especially c-Jun, JunB, and Fra-2; ref. 30). This is likely underpinned by activation of AP-1 factors by hypophosphorylated Rb, which in turn drives AP-1 members’ gene expression through a positive autoregulatory mechanism, leading to accumulation of AP-1 factors within the cell (30, 38, 39). Consistent with this, we observed that the reinstatement of Rb hypophosphorylation by INX-315 treatment of CDK4/6i–resistant cells increased expression of AP-1 family member genes (including *JUN*, *JUNB*, and/or *FOSL2*; Supplementary Fig. S12F). Furthermore, chromatin regions gaining accessibility in CDK4/6i–resistant cells treated with INX-315 were strongly enriched for AP-1 factor motifs and showed statistical overlap with publicly available AP-1 factor chromatin immunoprecipitation sequencing (ChIP-seq) datasets (Fig. 5F; Supplementary Fig. S12G). Moreover, genes proximal to regions of “opening” chromatin that also contained c-Jun–binding motifs were significantly upregulated after INX-315 treatment of resistant cells (Supplementary Fig. S12H). These data suggests that the senescence-defining chromatin state induced by CDK4/6i treatment of parental breast cancer cells is reinstated when proliferating, CDK4/6i–resistant cells are treated with CDK2 inhibition.

We next sought to determine whether specific biological processes linked to CDK4/6i–induced senescence in luminal breast cancer might be also regulated by enhancers activated during INX-315–induced senescence. First, we observed genes encoding for more than 30 different senescence-associated secretory phenotype (SASP) factors that were upregulated by CDK4/6i treatment of parental cells were also upregulated by INX-315 treatment of resistant cells (e.g., *IGBP3*, *VEGFA*, *HBEGF*, *TGFB1*, *TGFB2*; Fig. 5G). Also consistent with the effects of upfront CDK4/6 inhibition, genes associated with apoptotic evasion (*BCL2L1*), luminal differentiation (*KRT7*, *KRT8*), and glandular lumen morphogenesis (*CEACAM1*, *PRICKLE2*, *PARD6B*) were upregulated by INX-315 treatment of CDK4/6i–resistant MCF7 cells (Supplementary Fig. S13A–S13C). In many cases, these upregulated genes or gene sets were situated proximal to enhancers showing increased accessibility after INX-315 treatment (Fig. 5H).

### CDK2 Inhibition Prevents the Emergence of Acquired CDK4/6i Resistance

A key issue relevant to the clinical development of CDK2 inhibitors in luminal breast cancer is whether they might not only overcome acquired resistance to CDK4/6 inhibition, but also prevent it. To address this question *in vitro*, we treated parental MCF7 and T47D cells with control vehicle, abemaciclib, INX-315, or their combination. To model effective CDK4/6i therapy, we used a dose of abemaciclib (500 nmol/L) that induced potent cell cycle arrest (S-phase <1% of cells) at 7 days. The S-phase percentage remained low (<1%) in combination-treated cells, although more cells were arrested in G_2_–M compared with CDK4/6i monotherapy, possibly attributable to inhibition of CDK2/cyclin A or CDK1/cyclin B1 by INX-315 (Supplementary Fig. S14A).

**Figure 6.**
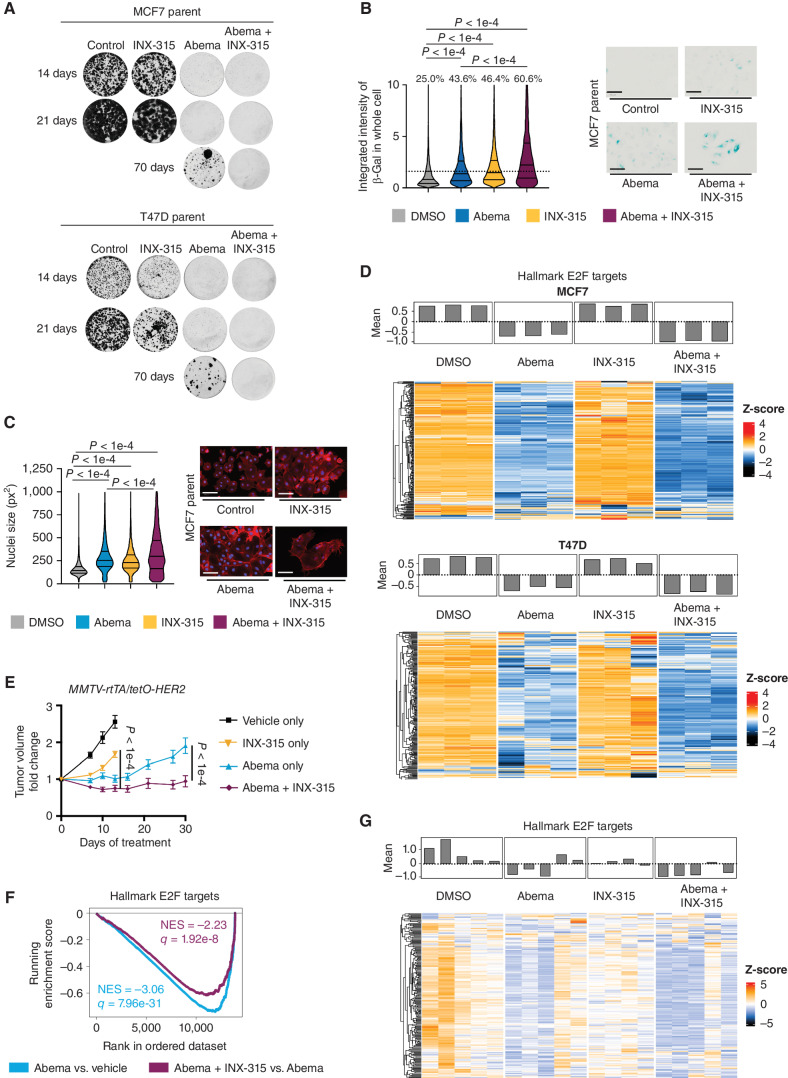
INX-315 delays the onset of acquired CDK4/6i resistance. **A,** Representative images from a clonogenic assay in which MCF7 and T47D cells were treated with control vehicle, abemaciclib (500 nmol/L), INX-315 (300 nmol/L for MCF7, 100 nmol/L for T47D), or the combination. Six technical replicates per condition. **B,** Representative images (right) and quantification (left) after treating MCF7 cells as in **A** for 7 days followed by staining for beta-galactosidase (β-gal) activity (scale bar = 100 µm). Note that representative images for control and abemaciclib group are identical to those used in Fig. 5A. **C,** Representative images (right) and quantification of relative nuclear size (left) for cells treated as in **B** (scale bar = 100 µm). Note that representative images of control and abemaciclib group are identical to those used in Fig. 5B. **D,** Heat map showing z scores for individual E2F target genes (RNA-sequencing) in MCF7 and T47D cells treated with drugs shown for 7 days at concentrations as in **A**. Bar plot above shows mean z-score for all genes in each sample. Three technical replicates per condition. **E,** Tumor growth curves for *MMTV-rtTA/tetO-HER2* tumors treated with control vehicle (*n* = 41 tumors in 5 mice), abemaciclib (*n* = 40 tumors in 5 mice), INX-315 (*n* = 39 tumors in 5 mice), or the combination (*n* = 29 tumors in 5 mice). Experiment was repeated twice. **F,** GSEA plots derived from RNA-sequencing performed on tumor tissue from experiment (**E**). Normalized enrichment score (NES) and q value (false discovery rate) were calculated as described in Methods. For **B** and **C**, *P* values calculated using unpaired *t* test; for **E**, error bars represent SEM; *P* values calculated using two-way ANOVA). **G,** Heat map showing z scores for individual E2F target genes (RNA-sequencing) in *MMTV-rtTA/tetO-HER2* tumors from **E**. Bar plot above shows mean z-score for all genes in each sample. Five samples for vehicle, abemaciclib, and combination; four samples for INX-315.

We then plated the same cells at low density and cultured them with the same agents over a period of ten weeks in a clonogenic assay. INX-315 monotherapy only slowed the growth of parental MCF7 and T47D cells modestly, consistent with it being a selective CDK2 inhibitor that spares CDKs 4 and 6. Consistent with the flow cytometry data, no differences were observed in the colony-forming assay between abemaciclib monotherapy and combined abemaciclib/INX-315 over the first 2 weeks. However, by week 8, we observed clear outgrowth of cells cultured in 500 nmol/L abemaciclib, consistent with acquired resistance, but not combination therapy. This difference was more clearly apparent by week 10 (Fig. 6A). We observed similar results in T47D cells and the luminal HER2-positive cell line BT474 when using 300 nmol/L palbociclib instead of abemaciclib (Supplementary Fig. S14B). This suggests that *in vitro*, concomitant CDK2 inhibition delays the emergence of resistance to CDK4/6i.

The fact that both CDK4/6i monotherapy and combined CDK4/6 + CDK2 inhibitor therapy caused a similar and near complete cell cycle arrest initially, but that resistance only emerged in the CDK4/6i monotherapy–treated cells, indicates that analysis of cell cycle profiles at early timepoints (e.g., 3–7 days) does not accurately predict long-term cell cycle control with cell cycle inhibitors. Rather, the duration of cell cycle withdrawal in response to senescence-inducing agents might be predicted by the “depth” of cell cycle arrest, indicated by the “intensity” of senescence biomarkers (40). Consistent with this notion, combined abemaciclib/INX-315 treatment for 7 days significantly increased indices of cellular senescence compared with abemaciclib alone, including the fraction of cells staining for β-galactosidase activity and nuclear size (Fig. 6B; Supplementary Fig. S6C). RNA sequencing showed that combination treatment for 7 days also suppressed the expression of E2F target genes more than abemaciclib alone. (Fig. 6D).

To determine the significance of these findings *in vivo*, we treated tumor-bearing *MMTV-rtTA/tetO-HER2* mice with vehicle control, abemaciclib, INX-315, or their combination. Consistent with our *in vitro* data, abemaciclib monotherapy and combined abemaciclib/INX-315 controlled tumor growth to a similar extent at early timepoints. However, although tumors developed resistance to abemaciclib by 3–4 weeks, this was not observed in tumors treated with combination therapy (Fig. 6E). Furthermore, analysis of tumor RNA at 7 days showed that the combination suppressed E2F target genes to a greater extent that abemaciclib alone (Fig. 6F and G). Thus, combined CDK4/6 + CDK2 inhibition induces a deeper suppression of E2F target genes associated with upregulation of some senescence biomarkers, and this in turn is associated with a more durable cell cycle arrest than treatment with CDK4/6i monotherapy.

## DISCUSSION

Despite a decades-long goal to identify selective CDK inhibitors, only a small number of compounds have successfully entered clinical practice, and all of these inhibit CDKs 4 and 6. Here, we describe the creation of a novel, potent, and selective inhibitor of CDK2 and characterize its activity in models of *CCNE1*-amplified cancer and CDK4/6i–resistant luminal breast cancer. Our findings strengthen the notion that CDK2 is a relevant and promising target for cancer therapy (6, 13, 41) and support the current efforts by Incyclix Bio, Pfizer, Blueprint Medicines, Incyte, and Allorion Therapeutics to develop CDK2 inhibitors in the clinic. They also answer important questions about CDK dependencies in different cancer types, the role of cell cycle plasticity as a driver of drug resistance in cancer, and the dynamic and partially reversible nature of CDK inhibitor–induced senescence.

Characterized by relative chemoresistance, genomic instability, and poor prognosis, *CCNE1-*amplified cancers represent an area of unmet need in oncology. Preclinical data suggest that these tumors proliferate in a CDK2-dependent manner (13, 19, 41). Our experimental data confirm and extend upon this concept by demonstrating that selective pharmacologic inhibition of the CDK2 kinase itself is sufficient to induce cell cycle arrest in these cancers. Moreover, their insensitivity to CDK4/6i indicates the primacy of CDK2 in driving the G_1_ to S transition in *CCNE1-*amplified cancer cells. This indicates that the “classical” model of G_1_ to S transition, wherein CDK4/6-mediated Rb phosphorylation is required to enable CDK2-driven entry into S-phase (1), does not apply to these tumors. This in turn implies that high levels of cyclin E can hyperactivate CDK2 to a level sufficient to overcome any absolute CDK4/6 requirement. Furthermore, our finding that CDK2 inhibitor monotherapy exerts durable control of tumor growth *in vivo* in *CCNE1-*amplified cancers (associated with persistent Rb hypophosphorylation and a reduction in cyclin A2) signifies that CDK4/6 activity does not rapidly compensate for CDK2 inhibition to restore proliferation, in contrast to what has been reported in nonmalignant cells (18). The implications of this are two-fold: first, it suggests that CDK2 inhibitor monotherapy (rather than combined CDK2/4/6 inhibition) should be the therapeutic strategy of choice for clinical trials in *CCNE1-*amplified tumors; second, it suggests that CDK2 inhibitors might have a wide therapeutic window in this setting, enforcing cell cycle arrest in tumor cells but not in other cells within the organism.

CDK4/6i–resistant breast cancer is a common and difficult clinical challenge, and a critical goal is to identify novel agents that will overcome this resistance by regaining control of the cancer cell cycle. Consistent with previous reports, our data suggest that combined CDK2 plus CDK4/6 inhibition might represent one such strategy (14). We show that although the proliferation of luminal breast cancers is initially CDK4/6-dependent, CDK2 activity can overcome persistent CDK4/6 inhibition, eventually restoring Rb phosphorylation to a level that facilitates cellular entry into S-phase. Importantly, by using a selective CDK2 inhibitor (rather than a combined CDK2/4/6 inhibitor), we have dissected out the contributions of different G_1_–S CDKs to the proliferation of resistant tumors and shown that restoration of cell cycle control requires both CDK2 inhibition and continuation of a CDK4/6i “beyond progression.” This suggests that the marked CDK2 inhibitor sensitivity of resistant tumors does not reflect a “switch” from CDK4/6 to CDK2 dependence. Rather, it reflects cell cycle plasticity, wherein the net contributions of CDK2 and CDK4/6 toward Rb phosphorylation ultimately drives a resistant cell into S-phase (18, 42). Importantly, our conclusions on CDK4/6i resistance are based upon cell models of resistance to both CDK4/6i monotherapy and combined CDK4/6 inhibition plus endocrine therapy. This is critical given that CDK4/6i are nearly always given with endocrine therapy in clinical practice. Furthermore, our novel in vivo model of acquired resistance to CDK4/6i is unique, being characterized by tumor response to continuous CDK4/6 inhibition before the eventual emergence of resistance in the absence of new driver mutations. This trajectory represents the most common clinical picture of acquired resistance and has been notoriously difficult to model in vivo.

Our studies in breast cancer have also uncovered two other notable features of cancer cell cycle biology. The first relates to the increasingly recognized phenomenon of cell cycle inhibitor–induced senescence. We have previously shown that CDK4/6i induced senescence is underpinned by chromatin remodeling that drives the transcriptomic hallmarks of the senescent phenotype (30). Here, we show that acquired resistance to CDK4/6i is associated with partial reversion to a “presenescent” chromatin state, and that addition of a CDK2 inhibitor in turn can reinstate the chromatin architecture of senescence. While the precise molecular differences between the senescence induced by CDK4/6i, CDK2 inhibitors, or their combination remain to be determined, our findings highlight that CDK inhibitor–induced senescence is reversible and dynamic. Moreover, therapeutic strategies aiming to specifically leverage the senescent state (e.g., combinations with senolytics or immunotherapy) must emphasize (i) holding cells in an arrested, senescent state and (ii) understanding differences in senescence induced by different CDK inhibitors in different cancer types. Second, we have found that although CDK4/6i monotherapy is sufficient to induce complete cell cycle arrest in breast cancer cells, their combination with CDK2 inhibitors upfront significantly delays the development of acquired resistance. The combination also induces greater suppression of E2F target genes, and we believe this reflects an underlying principle that the deeper these genes are suppressed below the threshold of proliferative arrest, the more difficult it is for a cancer cell to mount pro-proliferative forces strong enough to drive S-phase entry again. Importantly, our experiments also suggest that mechanisms of acquired CDK4/6i resistance cannot always be identified in short-term cell viability assays. Detailed preclinical studies comparing the benefits of upfront combination therapy versus sequential therapy are warranted.

INX-315 is currently being clinically investigated to determine its safety and efficacy in both *CCNE1*-amplified cancers as well as in CDK4/6i-resistant breast cancer (NCT05735080). In addition to clinical trials, we suggest several future preclinical studies that should be conducted with INX-315 and other selective CDK2 inhibitors. First, it will be important to identify biomarkers other than *CCNE1* amplification that are associated with response to CDK2 inhibitor monotherapy. Examples might include those that are correlated with high levels of cyclin E (e.g., loss of *FBXW7, MYC* amplification) or those indicative of DNA repair defects given the importance of CDK2 in activating numerous DNA repair factors (e.g., tumors with homologous recombination deficiency; ref. 5). Second, preclinical studies of rational combination therapies should be conducted to prioritize those that could be taken forward for clinical development. These include combinations with DNA damaging and/or cytotoxic chemotherapy, inhibitors of PARP, and possibly immune checkpoint inhibitors given recent reports that senescent cancer cells are immunogenic (25, 43, 44). Of particular importance will be the development of CDK2 + CDK4/6i combinations in the treatment of breast cancer. A potential concern here is that simultaneous inhibition of these three kinases might prove prohibitively toxic, and the results of trials combining INX-315 (and other CDK2 inhibitors) with CDK4/6i are eagerly awaited. Given our observation that CDK4/6-resistant breast cancer cells are markedly sensitive to the addition of INX-315 (IC_50_ <25 nmol/L), it is plausible that these combinations might be effective using low doses of selective CDK2 inhibitors, thereby improving the overall tolerability profile. Another combination worth exploring will be that of selective CDK2 plus novel CDK4 inhibitors (i.e., CDK6-sparing), with the rationale of reducing toxicity while exploiting the primary CDK4 dependency of many luminal breast cancers (e.g., NCT05262400; refs. 4, 19, 45). Finally, mechanisms of resistance to both CDK2 and combined CDK2/4/6 inhibition should also be explored in future work.

## METHODS

### Compounds

INX-315 was provided by Incyclix Bio. PF-07104091 was synthesized by PharmaAdvance. Abemaciclib and ribociclib were purchased from MedChemExpress. Palbociclib was purchased from Selleck Chemicals.

### Nanosyn CDK Biochemical *In Vitro* Assay

Compounds were tested in kinase assays (Nanosyn, Inc.). The assays were completed using microfluidic kinase detection technology (Caliper Assay Platform). The compounds were tested in 12-point dose–response format in singlicate at the *K*_m_ for ATP. Phosphoacceptor substrate peptide concentration used was 1 µmol/L and staurosporine was used as the reference compound for all assays. Details for individual Nanosyn assays performed are provided here:

### Microsomal Stability Assay

Microsomal stability was determined by Absorption Systems (Exton, PA). INX-315 was tested in mixed-gender human liver microsomes (Lot #1010420), male Sprague-Dawley rat liver microsomes (Lot #1910100), male CD-1 mouse liver microsomes (Lot #2010017), and male beagle dog liver microsomes (Lot #2010046) were purchased from XenoTech/BioIVT by Absorption Systems. The reaction mixture, minus NADPH, was prepared as described below. The test article was added into the reaction mixture at a final concentration of 1 µmol/L. The control compound, testosterone, was run simultaneously with the test article in a separate reaction. The reaction mixture (without cofactor) was equilibrated in a shaking water bath at 37°C for 5 minutes. The reaction was initiated by the addition of the cofactor, and the mixture was incubated in a shaking water bath at 37°C. Aliquots (100 µL) were withdrawn at 0, 10, 20, 30, and 60 minutes. Test article and testosterone samples were immediately combined with 400 µL of ice-cold 50/50 acetonitrile (ACN)/H2O containing 0.1% formic acid and internal standard to terminate the reaction. The samples were then mixed and centrifuged to precipitate proteins. All samples were assayed by LC-MS/MS using electrospray ionization. The peak area response ratio (PARR) of analyte to internal standard at each time point was compared to the PARR at time 0 to determine the percent remaining at each time point. Half-lives and clearance were calculated using GraphPad software (RRID:SCR_002798), fitting to a single-phase exponential decay equation.

### Modeling of INX-315 Docked to CDK2

Docking studies were performed to understand the binding interactions of INX-315 in the active site (ATP-binding site) of CDK2/cyclin E1. INX-315 was subjected to minimization using 1500 iterations, applied MMFF94 force field (used through the study), assigned Gasteiger charges, with an energy gradient convergence criterion of 0.0001 kcal/mol Å. The lowest energy conformation was determined. The crystal structure was downloaded from RCSB PDB (ID:1W98). Chain A was retained. Gasteiger charges were assigned. The 3D structure of CDK2 was prepared to fix all the defects and errors in the structures. Protein preparation includes addition of hydrogens, repair side chains, treat termini, fixing of atom type, protonation state, bond order, charges, and amides. It was minimized to remove any strain produced during earlier steps. Protomol was generated with a threshold of 0.5 and a bloat value of 1 Å, to generate an active site. INX-315 was docked into this active site using the Surflex-Dock GeomX (SFXC) method. The estimated binding affinity 10.840 for INX-315, (expressed as total_score which represents −llog*K*_d_), was reported. All the simulations were performed using SYBYL-X 2.1, and images were generated using Tripos Benchware 3D Explorer Viewer 2.7 (Certara Inc.).

### NanoBRET

Reaction Bio (Malvern, PA) conducted NanoBRET assays utilizing Promega NanoLuc technology with two replicates per experiment. The CDK2/E1 and CDK1/B1 values are the average of two separate experiments.

HEK293 cells were transfected using Opti-MEM without serum containing ratios of 9.0 µg/mL of Transfection Carrier DNA, 1.0 µg/mL of NanoLuc fusion vector DNA and 1 mL of Opti-MEM without phenol red. 30 µL of FuGENE HD transfection reagent was added to each milliliter of DNA mixture to form lipid:DNA complex. After incubation for 20 minutes at room temperature, 1 part lipid/DNA complex was added to 20 parts HEK293 cells in suspension. Cells were then plated and incubated overnight before being trypsinized and resuspended at 2 × 10^5^ cells/mL. NanoBRET tracer reagent was added to cells at a ratio of 1 part tracer to 20 parts cell suspension. Cells were added to a 384-well plate and incubated for 1 hour. Plates were removed and incubated at room temperature for 15 minutes to equilibrate before 3x complete substrate plus inhibitor was added. After 2–3 minutes of incubation at room temperature, donor and acceptor wavelengths (460 nm and 600 nm, respectively) were measured using an Envision 2104 plate reader.

### Biochemical Screen for Kinase Activity

INX-315 was first evaluated in the SelectScreen Biochemical Kinase Profiling at Thermo Fisher Scientific (Madison, WI). The primary screen consisted of INX-315 dosed at a single concentration of 100 nmol/L in 1% DMSO across each platform within the SelectScreen array of assays, LanthaScreen Eu Kinase Binding Assay; AdaptaScreen; and Z'LyteScreen using preestablished conditions at Thermo Fisher Scientific as shown in Supplementary Table S2. All target kinases that responded to greater than 90% inhibition in primary screen were retested as individuals for IC_50_ determination shown in Supplementary Fig. S1. The follow-up screen consisted of 10-point dose–response curves for INX-315 from 1 µmol/L to 0.0495 nmol/L.

### Animal Experiments

PDX models were performed by Crown Biosciences (OV5398, GA0103, and GA0114) or XenoSTART (ST4316B). Tumor fragments from stock mice were harvested and implanted subcutaneously into the flank of NOD/SCID mice (OV5398, RRID:IMSR_JAX:001303), BALB/c nude mice (GA0103 and GA0114, RRID:IMSR_RJ:BALB-C-NUDE), or athymic nude mice (Crl:NU(NCr)-Foxn1nu, RRID:IMSR_CRL:490) (ST4316B). For CDX models, female athymic nude mice (Crl:NU(NCr)-Foxn1nu, RRID:IMSR_CRL:490) were inoculated with 1 × 10^7^ 50% Cultrex OVCAR3 cells in the flank. Experiments performed using *MMTV-rtTA/tetO-HER2* mice were performed at the Peter MacCallum Cancer Centre. Tumor formation was induced and sustained in female mice with doxycycline as previously described (33). Randomization and treatment in all models began when median tumor volume reached a maximum of 300 mm^3^.

Mice were treated via oral gavage. INX-315 was prepared weekly in 100% PEG400; abemaciclib was prepared as previously described (33) and ribociclib (Selleckchem, S7440) was prepared weekly in 0.5% methyl cellulose. To generate abemaciclib-resistant *MMTV-rtTA/tetO-HER2* tumors, mice were treated continuously with abemaciclib at a dose of 75 mg/kg until tumors resumed growth as described in text. At this point and in all other *MMTV-rtTA/tetO-HER2* mice experiments, mice were randomized into treatment groups and abemaciclib was administered to indicated groups at a dose of 50 mg/kg. In all *MMTV-rtTA/tetO-HER2* mice experiments, INX-315 was administered at a dose of 50 mg/kg twice daily. For PDX model ST4316B, ribociclib was administered at 50 mg/kg per day.

Studies with *MMTV-rtTA/tetO-HER2* mice were performed in compliance with federal laws and institutional guidelines as approved by the Animal Ethics Experimentation Committee of the Peter MacCallum Cancer Centre. Studies with GA and OV5398 models were performed in compliance by the Institutional Animal Care and Use Committee (IACUC) of CrownBio and in accordance with the regulations of the Association for Assessment and Accreditation of Laboratory Animal Care. Studies with OVCAR3 CDX models were performed in compliance with IACUC of Champions Oncology (Hackensack, NJ) and studies with ST4316B were performed in compliance with IACUC of XenoSTART.

### Cell Lines, IC_50_ Determination, and *In Vitro* Experiments

Cell lines were obtained from ATCC, Xenotech, or Sigma-Aldrich (2019–2021). All media were supplemented with 10% FBS. Ovmana (JCRB1045, RRID:CVCL_3111), OVKATE (JCRB1044, RRID:CVCL_3110), and OVCAR3 (HTB-161, RRID:CVCL_0465) were cultured in RPMI1640 medium (Gibco, 11875–093) with OVCAR3 also supplemented with 1× Insulin-transferrin-selenium (ITS, Thermo Fisher Scientific, 41400045) and 1× Glutamax (Gibco, 35050061). MKN1 (RRID:CVCL-1415), Kuramochi (JCRB0098, RRID:CVCL_1345), MCF7 (HTB-22, RRID:CVCL_0031), and T47D (HTB-133, RRID:CVCL_0553) were cultured in RPMI1640 medium (Gibco, 11875–093) supplemented with 1× Glutamax. Media for MCF7 and T47D was also supplemented with 1× HEPES (Gibco, 15630080). CAOV3 (#HTB-75, RRID:CVCL_0201) and OAW28 (Sigma-Aldrich, 85101601, RRID:CVCL_1614) were cultured in DMEM (Gibco, 1195–065) with OAW28 supplemented with 1 mmol/L sodium pyruvate (Gibco, 11360070) and 20IU/I bovine insulin (Sigma-Aldrich, I6634). COV318 (07071903, RRID:CVCL_2419), Hs68 (CRL-1635, RRID:CVCL_0839), and BT474 (HTB-20, RRID:CVCL_0179) were cultured in DMEM supplemented with 1× Glutamax, with medium for BT474 also supplemented with 1× ITS. PA-1 (CRL-1572, RRID:CVCL_0479) cells were cultured in Eagle minimum essential medium (EMEM, Gibco, 11965) supplemented with 1× Glutamax (Gibco, 35050061). SKOV3 (HTB-77, RRID:CVCL_0532) cells were cultured in McCoy 5a medium modified (Gibco, 16600082). MCF7PDRCl5–1 (palbociclib-resistant MCF7 cells, G1 Therapeutics) were cultured in EMEM (Gibco, 11965) supplemented with 1 × Glutamax, 1 × ITS, 1 µmol/L PD0332991 (Selleckchem, S1116). FUOV-1 (DSMZ ACC 444, RRID:CVCL_2047) were cultured in DMEM/F-12 (Gibco, 11320–033) supplemented with 1× Glutamax.

All cell lines were routinely tested for *Mycoplasma* once a month and within a month of conducting experiments using via either PCR testing or InvivoGen's MycoStrip detection kit (rep-mysnc-100). All cell lines were authenticated using short tandem repeat profiling and used for experiments no sooner than two passages after thawing and no more than five passages after thawing.

MCF7 abemaciclib-resistant cells were generated by continuous treatment with abemaciclib at a concentration of 250 nmol/L for 72 days, followed by 500 nmol/L for 48 days. MCF7 fulvestrant-resistant cells were generated by continuous treatment of 100 nmol/L Fulvestrant for 125 days. MCF7 abemaciclib/fulvestrant-resistant cells were generated by treating abemaciclib-resistant cells continuously with 100 nmol/L fulvestrant for 137 days. T47D abemaciclib-resistant lines were generated by continuous treatment with abemaciclib at a concentration of 500 nmol/L for 73 days. T47D fulvestrant–resistant cells were generated by continuous treatment with 100 nmol/L fulvestrant for 68 days. T47D abemaciclib/fulvestrant-resistant cells were generated by treating abemaciclib-resistant cells with continuous treatment of 100 nmol/L Fulvestrant for 60 days. Resistant cell lines were cultured in medium containing drug(s) that the line is resistant to and further authenticated using short tandem repeat profiling.

OVCAR3, COV318, Kuramochi, MKN1, SKOV3, MCF7PDRCl5–1, FUOV-1, Ovmana, PA-1, OVKATE, OAW28, and CAOV4 cells were plated in 96 well plates and treated with a ten-point dose concentration from 10 µmol/L to 0.3 nmol/L of INX-315 PF-07104091, or palbociclib for 6 days. An exception to this is MCF7PDRCl5-1 cells which were first cultured for 5 days without palbociclib then plated as described. MCF7PDRCl5-1 cells were treated with either a dose curve of the indicated CDK4/6i or a dose curve of INX-315 + 1 µmol/L of the indicated CDK4/6i. Cell viability was determined using the CellTiter-Glo assay (Promega, G9241) following manufacturer's instructions. Plates were processed on either BioTek Synergy2 multi-mode (Agilent) or ClarioSTAR (BMG Labtech) plate readers. T47D and MCF7 cells were seeded into 364-well plates using BioTek 406 (Aglient) and treated with a ten-point dose concentration from 10 µmol/L to 0.3 nmol/L of INX-315 for 2× doubling times. Plates were fixed with 4% PFA and stained with Rhodamine-Phalloidin (Bio-Trend, 00027) and DAPI (5 mg/mL, Invitrogen, catalog no. D1306). Plates were imaged using CellInsight CX7 LZR and DAPI-positive cells were counted using Thermo Fisher Scientific HCS Studio Cell Analysis Software.

For *in vitro* experiments outlined below, T47D and MCF7 parent and resistant lines were treated for 7 days. Where indicated, both cell lines were treated with 500 nmol/L of abemaciclib and for INX-315, T47D was treated with 100 nmol/L and MCF7 with 300 nmol/L. All other cell lines were treated for 24 hours with concentrations of INX-315 or PF-07104091 as indicated, except for RNA-sequencing experiments using OVCAR 3 cells where treatment was for 7 days.

### Western Blotting

Western blotting was performed as previously described (30) with slight modifications. Cell lysates (T47D and MCF7) were extracted with Cell Lysis Buffer (Cell Signaling Technology, 9803) containing Complete mini EDTA-free protease inhibitor cocktail and PhosSTOP. Alternatively, all other cell lysates were collected using RIPA buffer (Thermo, 89901) + Halt Protease/Phosphatase Inhibitors (Thermo Fisher Scientific, 1861280) followed by cell scraping. Tumor fragments were crushed using liquid nitrogen followed by lysis with RIPA buffer. Samples were normalised by total protein concentration measured using a DC assay or similar. 20–30 µg of protein or 500,000 directly lysed cells (for lysates normalized to cell number) were mixed with Sample Buffer (Invitrogen, NP0008) or Laemmli buffer (Bio-Rad, 1610747) + 5% BME and heated at 70–95°C for 10–12 minutes before being run on 4%–12% gradient or 15% gels (Invitrogen, NP0335, Bio-Rad, catalog no. 1610159). Antibodies used for detection include total Rb (BD, 554136, RRID:AB_395259; Cell Signaling Technology, 9309S, RRID:AB_823629), phospho- (Ser807/811; Cell Signaling Technology, 8516, RRID:AB_11178658), cyclin A2 (Cell Signaling Technology, 4656S, RRID:AB_2071958), GAPDH (Cell Signaling Technology, 97166S, RRID:AB_2756824), cleaved PARP (Cell Signaling Technology, 9541, RRID:AB_331426), Bcl-xL (Abcam, ab32370, RRID:AB_725655), Bcl-2 (Abcam, ab182858, RRID:AB_2715467), cyclin E1 (D7T3U; Cell Signaling Technology, 20808, RRID:AB_2783554), (phospho-CDC6 (Abcam, ab75809, RRID:AB_1310068), CDC6 (Cell Signaling Technology, 3387, RRID:AB_2078525), phospho-nucleolin (Abcam, ab155977, RRIB:AB_3075493), nucleolin (CST, 87792, RRID:AB_2800106) and vinculin (Sigma, v9131, RRID:AB_477629). Western blot images were acquired on the Odyssey CLx Imaging System (LI-COR Biosciences) or ChemiDoc MP Imaging System (Bio-Rad) using Image Studio Software.

### Cell Cycle Flow Cytometry with BrdU/EdU

One hour prior to endpoint, either 10 µmol/L BrdU or EdU was applied to the cells. Cells were stained using the Near-IR Fixable Viability Stain (Invitrogen, L34976) for 20 minutes at room temperature. Cells were fixed and permeabilized using FoxP3/Transcription Factor Staining Buffer Set (eBioscience, 00–5523–00; for BrdU-treated cells) or Click-iT fixative (for EdU-treated cells). DNA was denatured using 2N HCl + 0.5% (v/v) Triton X-100 for 30 minutes. The acid was neutralized using 0.1 mol/L Na_2_B_4_O_7_.10H_2_O (pH 8.5) followed by 0.5% BSA in PBS. Cells were then stained with BrdU antibody (clone 3D4, BioLegend, 364118, RRID:AB_2814318) for 1 hour. The EdU Click-iT reaction was performed using a Click-iT EdU Flow Cytometry Assay Kit (Invitrogen, C10418 or C10425). Prior to acquisition on the BD A3 Symphony or FACSCelesta, DNA was stained using FxCycle Violet ReadyFlow Reagent (BrdU) or FxCycle Far Red nucleic acid stain (Invitrogen, F10348) in combination with Rnase (Sigma R4642) (for EdU-treated cells). A minimum of 10,000 live cell events were recorded for each sample and data was analyzed using Flow Jo (Tree Star, RRID:SCR_008520).

### B-Galactosidase Staining

Cells were seeded into chamber slides (Ibidi, 80841) and treated with DMSO, INX-315 and/or abemaciclib as indicated. Cells were fixed and stained using Senescence Detection Kit (Abcam, ab65351) and incubated at 37°C overnight in CO_2_ free incubator. Slides were counterstained with Rhodamine-Phalloidin (Bio-Trend, 00027) and DAPI (5 mg/mL, Invitrogen, D1306). Analysis of fluorescence images (DAPI, Phalloidin) and beta-galactosidase staining (β-Gal; brightfield) was conducted using CellProfiler (Broad Institute of MIT and Harvard, version 4.1.3; https://cellprofiler.org/releases/). In brief, after identification of “nuclei” in the DAPI channel as primary objects, we added a filter step to exclude everything with an area <20 px2 from further analysis (likely cell debris). For subsequent identification of secondary objects, we used the phalloidin channel to propagate the area of the “cell” around each “nucleus”. This cell area was used to create a mask and β-Gal intensity was measured as IntegratedIntensity (i.e., sum of the pixel intensities within the cell object) in the inverted beta-Gal image inside this cell mask. Finally, “MeasureObjectSizeShape” modules were used to quantify the areas of the “cell” and “nuclei” populations. For MKN1 and OVCAR3 cells, the cutoff for integrated β-Gal intensity was set at the 75th percentile of the control population (920 for OVCAR3 and 460 for MNK1). The percentage of β-Gal–positive cells was then calculated by dividing the number of cells with an intensity above this cut-off by the number of all cells

### Determination of Impact of *CCNE1* Knockdown on Cell Cycle in CDK4/6i–Resistant Cells

For siRNA-mediated gene silencing of *CCNE1*, cells were forward transfected with siRNA ON-TARGETplus (Dharmacon, GE Life Sciences) against human CCNE1 (ON-TARGETplus Human CCNE1 (898) siRNA – J-003214–10) or OnTarget Plus non-targeting (OTP-NT) siRNA using DharmaFECT-3 (Dharmacon, GE Life Sciences). Cells were seeded at a density of 5.0 × 10^5^ cells in 145-mm dishes. At 24 hours post seeding, siRNAs were complexed with DharmaFECT-3 in OptiMEM (Gibco) for 45 minutes before being added dropwise for a final concentration of 10 nmol/L siRNA. At 24 hours post transfection, fresh media were supplemented. Cells were collected for protein and cell cycle analysis 5 days after transfection.

### Colony Formation Assay

For prevention of resistance experiments with palbociclib treatment, T47D and BT474 cells were plated at 10,000 cells/well and 25,000 cells/well, respectively, in 6-well plates. For prevention of resistance experiments with abemaciclib treatment, T47D and MCF7 parent cells were plated at 2,000 cells per well in 6-well tissue plates. For MCF7-resistant cell lines treated with INX-315 or PF-07104091, cells were plated at 30,000 cells/well in 6-well plates, and treated with 300 nmol/L of respective CDK2 inhibitor along with abemaciclib and/or fulvestrant. Plates were treated with indicated drug treatments with medium and treatments refreshed every 3–7 days. Plates were fixed and stained with crystal violet (0.01%) in 1% methanol for 1 hour. Plates were washed with water and dried before scanning on either EPSON V600 scanner or Bio-Rad ChemiDoc MP.

### IHC

*MMTV-rtTA/tetO-HER2* tumors were stained with antibodies against Ki-67 (Biocare Medical, CRM325, RRID:AB_2721189), phospho-Rb Ser807/811 (Cell Signaling Technology, 8516, RRID:AB_11178658) or total Rb (Abcam, ab181616, RRID:AB_2848193). Images were acquired with a Nikon Eclipse E600 microscope, and three to four fields were analyzed per tumor. Image analysis was performed using a semi-automated in-house platform (ImageJ, RRID:SCR_003070).

### RNA Sequencing

RNA was extracted from cell pellets or crushed tumor pieces using NucleoSpin RNA plus kit (Macherey-Nagel, 40955) as per manufacturer’s protocol. For cell line studies, all studies were performed with biological triplicates. For tumor tissue, sample number is shown in each figure. Libraries were prepared using QuantSeq 32 mRNA-Library Prep Kit FWD (Lexogen). RNA quantity and quality were assessed on TapeStation 4150 (Agilent). Reads were sequenced on NextSeq500 (Illumina) using 75-cycle High Output Reagent kits. Fastq files were trimmed for adaptors and poor-quality reads using BBDuk (v38.00). The trimmed reads were aligned to either the hg38 or mm10 reference genome using STAR (v2.7.5, RRID:SCR_004463; ref. 46). For PDX samples, mouse reads were filtered out using XenofilteR (v1.6; ref. 47). Read counts were generated using featureCounts (v2.0.1, RRID:SCR_012919) with annotations from GENCODE (gencode.vM25.basic.annotation.gtf for mouse and gencode.v35.annotation.gtf for human; ref. 48). Gene expression values were normalized to Counts Per Million (CPM) using sizefactors from the DESeq2 package (version 1.36.0, RRID:SCR_000154; ref. 49). Differential gene expression analysis was performed using DESeq2. Gene set enrichment analysis (GSEA) was conducted using the clusterProfiler R package (v4.4.4, RRID:SCR_016884; ref. 50). In this analysis, a preranked list of genes was generated on the basis of the shrunken fold change obtained through the Ashr shrinkage algorithm. The enrichment score for each gene set of interest was calculated by accumulating a running sum. Single-sample GSEA (ssGSEA) was performed using the GSVA package (51). This analysis was performed by using the normalized log_2_-transformed gene expression matrix. For each individual sample, an enrichment score was calculated by applying a weighted running sum to the normalized ranking scores of genes within the gene set. Predefined gene sets were sourced from the Molecular Signatures Database (Hallmark, C2, C5; refs. 52, 53) using the msigdbr package (version 7.5.1). Heat maps were generated using ComplexHeatmap (54) and gene clustering was carried out via hierarchical clustering using Euclidean distance and complete linkage. For generating senescence scores, gene sets were obtained from Jochems and colleagues (28). SASP-related genes were obtained from Wang and colleagues (55). *P*_adj_ values for gene expression plots were derived from the Deseq2 output. *P* values for ssGSEA scores were determined using *t* tests.

### ATAC Sequencing

Omni-ATAC (56) was performed on either fresh or cryopreserved cells, using 50,000 nuclei per sample with slight deviations from the manufacturer’s instructions. All samples were run with biological duplicates. Deviations included the use of Illumina Tagment DNA enzyme and Buffer (Illumina, 20034197). qPCR side-reactions were performed to determine optimal PCR cycle number for each sample. Libraries were assessed using the TapeStation D1000 Assay and sequenced on the Illumina NextSeq 500 (75-bp paired-end reads, approximately 30–40 million paired-end reads per sample). Fastq files were trimmed for adaptors and poor-quality reads using BBDuk (version 38.00). Trimmed reads were aligned to hg38 reference genome using BWA-MEM (v0.7.17). Duplicate reads, blacklisted reads, chrM aligned reads, and multimapping reads were filtered out using picard (v3.0.0; http://broadinstitute.github.io/picard/, RRID:SCR_006525), samtools (v1.9, RRID:SCR_002105; ref. 57) and bedtools (v2.27.1, RRID:SCR_006646; ref. 58). Peak calling was performed using Genrich with parameters “-a 100 -q 0.05 -g 1.” Differential accessibility analysis was carried out using DiffBind (v3.6.1; refs. 59, 60). Peaks were annotated into genomic regions using ChIPseeker (v1.32.0; ref. 61). Motif enrichment analysis was performed using HOMER (v4.11; ref. 59) using parameters “-size 200 -len 6,10,15,20 -p 10.” GSEA was conducted using ChIP-Enrich (v2.20.0; ref. 62). FIMO (v5.4.1) was used to identify Jun-binding motifs. Cistrome DB Toolkit (http://dbtoolkit.cistrome.org/) was used to identify regulators that potentially bind to open chromatin (63). BETA (v1.0.7; ref. 64) was used to integrate chromatin accessibility data with differential gene expression data to infer activating function.

### Statistical Analyses

Statistical analysis was performed as described for each experiment. Two-way ANOVA was used for the analysis of tumor growth curves. All statistical tests were two-sided. Differences were considered statistically significant at a *P* value of less than 0.05.

### Data Availability Statement

The next-generation sequencing data in this study is publicly available in Gene Expression Omnibus (GEO, RRID:SCR_005012), accession number GSE243154. We will provide reviewers with links to this data upon request. All code used to generate results and figures for this paper can be found at https://github.com/Goel-Laboratory/2023_CDK2i_paper.

## Supplementary Material

Supplementary Synthetic Methods Description of methods to synthesize INX-315

Supplementary Table 1 Experimental conditions for Nanosyn assays

Supplementary Table 2 LanthaScreen Eu Kinase Binding and Z’-Lyte Kinase Assay data for INX-315

Supplementary Figures 1-14 Supplementary Figure 1: Characterization of INX-315.Supplementary Figure 2: Effect of INX-315 and PF-07104091 on viability of cancer and non-malignant cell lines.Supplementary Figure 3: Effect of INX-315 on cell cycle in CCNE1-amplified cancer cell lines.Supplementary Figure 4: Further characterization of the effects of INX-315 in in vitro and in vivo models of CCNE1-amplified cancer.Supplementary Figure 5: Effects of INX-315 treatment on gene expression in models of CCNE1amplified ovarian carcinoma.Supplementary Figure 6: Characterization of CDK4/6 inhibitor-resistant cell lines.Supplementary Figure 7: Response of CDK4/6 inhibitor-resistant breast cancer cells to INX-315 and PF-07104091.Supplementary Figure 8: Effects of INX-315 and PF-07104091 on CDK4/6 inhibitor-resistant breast cancer cells.Supplementary Figure 9: Impact of INX-315 +/- continued CDK4/6 inhibition in CDK4/6 inhibitorresistant breast cancer cells.Supplementary Figure 10: Characterization of a new mouse model of acquired CDK4/6 inhibitor resistance.Supplementary Figure 11: Effect of INX-315 on expression of senescence-related genes in CDK4/6 inhibitor-resistant breast cancer cells.Supplementary Figure 12: Epigenomic and transcriptomic features of INX-315 induced senescence in CDK4/6 inhibitor-resistant breast cancer.Supplementary Figure 13: Impact of INX-315 treatment of CDK4/6 inhibitor resistant breast cancer on expression of apoptosis and differentiation-related genes.Supplementary Figure 14: Impact of co-inhibition of CDK2 and CDK4/6 on cell cycle and development of CDK4/6 inhibitor resistance in breast cancer cells.
