# Correction: INX-315, a Selective CDK2 Inhibitor, Induces Cell Cycle Arrest and Senescence in Solid Tumors

**DOI:** 10.1158/2159-8290.CD-25-1502

**Published:** 2025-10-06

**Authors:** Catherine Dietrich, Alec Trub, Antonio Ahn, Michael Taylor, Krutika Ambani, Keefe T. Chan, Kun-Hui Lu, Christabella A. Mahendra, Catherine Blyth, Rhiannon Coulson, Susanne Ramm, April C. Watt, Sunil Kumar Matsa, John Bisi, Jay Strum, Patrick Roberts, Shom Goel

In the original version of this article (1), an error was introduced by the compositor in the CDK/cyclin B1 pairing in Fig. 1D and should read: CDK1/cyclin B1. Figure 1D has been corrected in the latest online HTML and PDF versions of the article. The publisher regrets the error.
