# Supplementary material for: INX-315, a Selective CDK2 Inhibitor, Induces Cell Cycle Arrest and Senescence in Solid Tumors
Source: Cancer Discov. 2023 Dec 1;14(3):446–67. doi: 10.1158/2159-8290.CD-23-0954 (PMC10905675; doi:10.1158/2159-8290.CD-23-0954)
Supplement: Supplementary Synthetic Methods — Description of methods to synthesize INX-315 [file cd-23-0954_supplementary_synthetic_methods_suppsm.pdf]

INX-315 Synthetic Chemistry Experimentals and Process as related to Figure 1 and Supplementary Figure 1

General Scheme: 4-((3'-oxo-2',3'-dihydro-1'H-spiro[cyclohexane-1,4'-pyrimido[5',4':4,5]pyrrolo[2,1-c][1,2,4]triazin]-7'-yl)amino)benzenesulfonamide

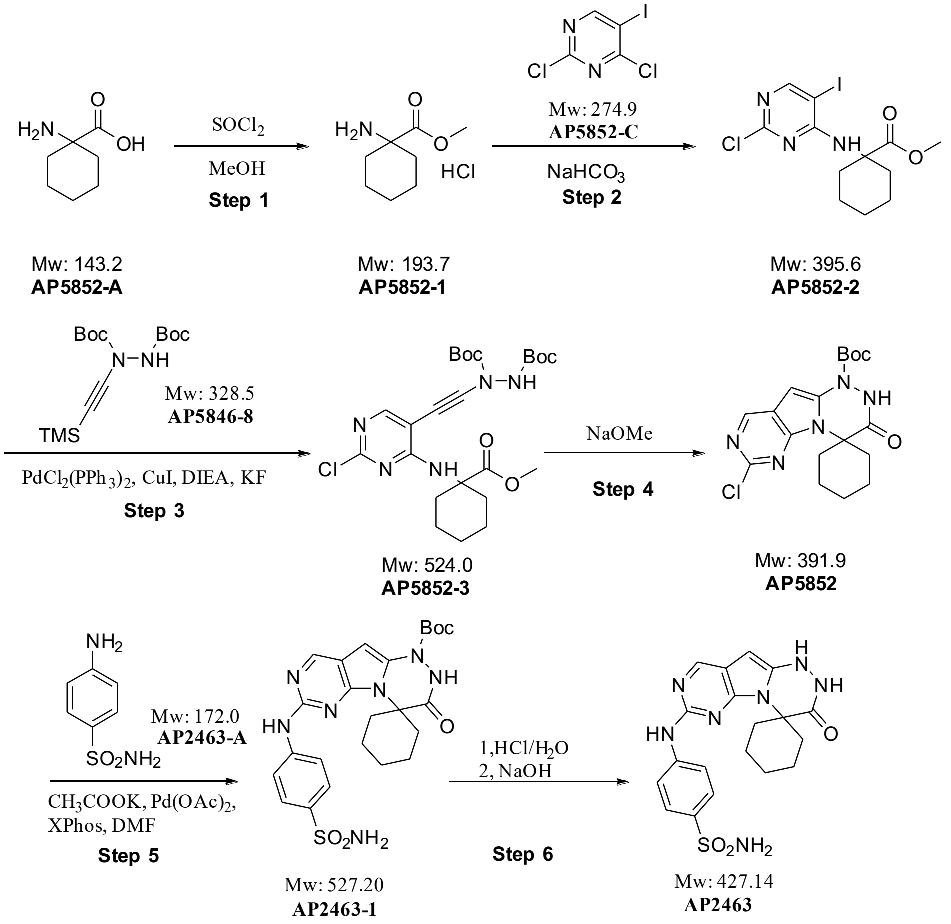

Experimental Details

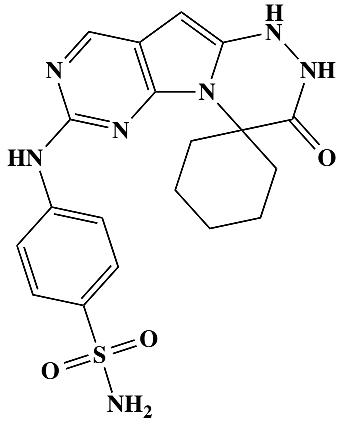

4-((3'-oxo-2',3'-dihydro-1'H-spiro[cyclohexane-1,4'-pyrimido[5',4':4,5]pyrrolo[2,1-c][1,2,4]triazin]-7'-yl)amino)benzenesulfonamide

Step 1:

To a solution of **AP5852-A** (6.5 kg, 45.46 mol) in MeOH (32.5 L) at 5 °C, $SOCl_2$ (8.11 kg, 68.18 mol, 1.5 eq) was added slowly. The reaction mixture was heated to reflux for 17 h and then concentrated to remove $SOCl_2$ and MeOH. The residue was heated to reflux in ethyl acetate (39 L) for 0.5 h and cooled to 15 °C. The solid was collected by filtration, washed with ethyl acetate (6.5 L), and then dried at 45 °C under vacuum overnight to give off-white solid, **AP5852-1**, 7.4 kg, 84% yield. LCMS (ESI+): m/z 158.3 (M+H); ^{1}H NMR (400 MHz, DMSO) δ 8.70 (s, 3H), 3.78 (s, 3H), 1.94-2.01 (m, 2H), 1.77-1.83 (m, 2H), 1.65-1.71 (m, 2H), 1.53-1.57 (m, 2H), 1.43-1.46 (m, 2H)

Step 2:

To a solution of **AP5852-C** (6.6 kg, 25.5 mol, 1.0 eq) and **AP5852-1** (5.6 kg, 30.6 mol, 1.2 eq) in DMF (3.2 L) and MeCN (33.2 L) at RT, $NaHCO_3$ (8.1 kg, 101.9 mol, 4.0 eq) was added. The mixture was heated to 80 °C for 3 days and then cooled to 30 °C. The solid was filtered off and the filtrate was concentrated. The residue was dissolved in MTBE (48 L), washed with water (9.6 L), 10% Citric acid (9.6 L) and brine (9.6 L). The organic layer was filtered through a pad of silica gel (200 g). The filtrate was concentrated under vacuum. The residue was heated to 45 °C in heptane (48 L) and THF (4.8 L) for 1 h and then cooled to -15 °C. The solid was collected by filtration, washed with heptane (3 L), and dried at 35 °C under vacuum overnight to give pale yellow solid, **AP5852-2**, 6.6 kg, 70% yield. ^{1}H NMR (400 MHz, DMSO) δ 8.47 (s, 1H), 6.49 (s, 1H), 3.61 (s, 3H), 2.15-2.18 (m, 2H), 1.81-1.87 (m, 2H), 1.33-1.62 (m, 6H)

Step 3:

To a solution of **AP5852-2** (5.5 kg, 13.9 mol, 1.0 eq), AP5846-8 (5.48 kg, 16.7 mol, 1.2 eq), DIPEA (5.38 kg, 41.7 mol, 3.0eq), $PdCl_2(PPh_3)_2$ (195.2 g, 0.278 mol, 0.02 eq), CuI (66.5 g, 0.348 mol, 0.025 eq) in DMF (22 L) at RT under N_2, KF (24.2 kg, 41.7 mol, 3.0 eq) was added. The mixture was heated to 45 °C for 4 h and then cooled to 5 °C. The reaction mixture was quenched with 44 L of ice-water and 1.1 L ammonium hydroxide, extracted with MTBE (38 L). The organic layer was washed with water (22 L x 2). Active carbon (550 g) and Na_2SO_4 (2.7 kg) were added into the organic solution at RT for 1h. The solid

was filtered off over a pad of silica gel (2.7 kg). The filtrate was concentrated. The residue was dissolved in MeOH (16.5 L), which was used in the next step directly. LCMS (ESI+): m/z 524.4/526.4 (M+H).

Step 4:

To the solution of **AP5852-3** in MeOH at 0 °C, 40% NaOMe (2.25 kg, 12.51 mol, 0.9 eq) was added. The reaction mixture was heated to 20 °C overnight and then was cooled to 0 °C. The solid was filtered off and the filtrate was concentrated. The residue was agitated in ethyl acetate (27.5 L) and water (27.5 L) at 20 °C for 6 h. The solid was collected by filtration, washed with water (5.5 L) and ethyl acetate (5.5 L), and then dissolved in acetone (51 L) at 50 °C. The solution was cooled to 10 °C. The solid was collected by filtration, washed with acetone (17 L), and dried at 45 °C under vacuum overnight to give off-white solid, **AP5852**, 3.6 kg, 66% yield for these 2 steps. LCMS (ESI+): m/z 392.2/394.2 (M+H); ^{1}H NMR (400 MHz, DMSO) δ 8.76 (s, 1H), 6.04 (s, 1H), 3.61 (s, 3H), 2.25-2.30 (m, 2H), 1.89-2.00 (m, 6H), 1.74-1.77 (m, 1H), 1.27-1.57 (m, 1H)

Step 5:

To a solution of **AP5852** (50.0 g, 127.5 mmol) and **AP2463-A** (26.3 g, 153.0 mmol, 1.2 eq.) in DMF (0.75 L, 15 V) under N_2, AcOK (18.8 g, 191.0 mmol, 1.5 eq.), $Pd(OAc)_2$ (0.86 g, 3.8 mmol, 0.03 eq.) and XPhos (5.15 g, 16.3 mmol, 0.064 eq.) were added. The resulting mixture was stirred at 80 °C for 16 h. Inorganic salt was filtered off over a pad of Celite (25 g) at 60~80 °C, and the filtrate was charged to water (1.5 L, 15 V) at 30-40 °C. After 0.5 h at 25 °C, the solid was collected by filtration. The wet cake (about 200 g) was dissolved in DMF (0.75 L, 15 V) at 75 °C. Si-thiol (6.5 g) was added. After 1 h at 75 °C, activated carbon (3.25 g) was added. After additional 1 h at 75 °C, the solid was filtered off. The filtrate was charged to water (0.75 L, 15 V) at 35 °C. After 0.5 h at 35 °C, the solid was collected by filtration, washed with water (1.5 L), and dried at 50°C under vacuum overnight to give pale yellow solid, **AP2463-1**, 64.0 g; 92.5% pure by HPLC. The crude **AP2463-1**(64 g.0) was heated in the mixture of isopropanol (150 mL) and Ethyl acetate (30 mL) at 75 °C for 1 h and then cooled down to 20 °C. The solid was collected by filtration, washed with the mixture of isopropanol (150 mL) and Ethyl acetate (30 mL) at 75 °C, and then dried at 50°C under vacuum overnight to give pale yellow solid, **AP2364-1**, 56.0 g, 83% yield. LCMS (ESI+): m/z 528.4 (M+H); ^{1}H NMR (400 MHz, DMSO) δ 10.17 (s, -1H), 9.83 (s, 1H), 8.62 (s, 1H), 8.00 (d, J = 8.9 Hz, 2H), 7.74 (d, J = 8.8 Hz, 2H), 7.15 (s, 2H), 5.81 (s, -1H), 2.29 – 2.48 (m, 1H), 2.22 – 1.76 (m, 3H), 1.49 – 1.65 (m, 9H),1.06 (d, J = 6.1 Hz, 6H).

Step 6:

To the solution of concentrated HCl (750 mL, 15 V) at 10 °C, **AP2463-1** (50.0 g, 19.0 mmol) was added in portions. The resulting mixture was stirred at 5 °C for 1 h. EtOH (1.5 L) was added. The solid was collected by filtration and washed with EtOH (50.0 mL*3, 5 V*3). The cake (about 70 g; 97.97% pure by HPLC) was dissolved in DMF (750 mL) under N_2. 5% Na_2CO_3 (100 mL) was added. The mixture was stirred at 5 °C for 0.5 h. Si-thiol (4.0 g) was added. After 0.5 h at 5 °C, activated carbon (2.0 g) was added. After 0.5 h at 5 °C, the solid was filtered off, and the filtrate was added to water (1500.0 mL). After 3 h at 55 °C, the solid was collected by filtration, washed with water (500.0 mL), and dried under vacuum at 50°C overnight. The solid (33.0 g; 97.82% pure by HPLC) was dissolved in DMSO (250 mL) at 25 °C under N_2, and then added to water (250.0 mL) at 25 °C. After 0.5 h at 25 °C, the solid was collected by filtration, washed with water (500.0 mL), and dried under vacuum at 50 °C overnight to give off-white solid, **AP2364 (INX-315)**, 31.0 g, 76% yield. LCMS (ESI+): m/z 428.4 (M+H); ^{1}H NMR (400

MHz, DMSO) δ 9.80 (s, 1H), 8.58 (s, 1H), 8.02 (d, *J* = 8.8 Hz, 2H), 7.77 (d, *J* = 8.8 Hz, 2H), 7.20 (s, 2H), 5.78 (s, 1H), 5.44 (s, 2H), 2.30 (t, *J* = 11.5 Hz, 2H), 2.17 – 1.91 (m, 6H), 1.82 (d, *J* = 5.2 Hz, 1H), 1.60 (d, *J* = 9.3 Hz, 1H).

LCMS spectrum of INX-315

1. LC-MS method

**Instrument:**	Waters 2695- ZQ 2000
**Column：**	Agilent SB-C18 4.6*50 mm, 5um
**Detector：**	2996 PDA
**Column Temperature:**	30 ℃
**Detection Wavelength:**	254/220 nm
**Mobile phase:**	A: HPLC grade H_2O (0.1% Formic acid)<br>B: HPLC grade Methanol
**Flow rate:**	1.0 mL/min
**Ion Mode：**	ESI
**Voltages：**	Capillary: 3.0 kV<br>Cone: 25 V<br>Extractor: 3 V
**Source Temp:**	120 °C

Time table

Time (mins)	%B
0	20
2.0	90
4.0	90
4.1	20
6.5	20

2. LCMS spectrum of Int-5 (batch# 3857-005-P1)

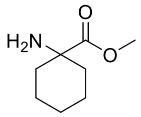

5

Molecular Weight: 157.21

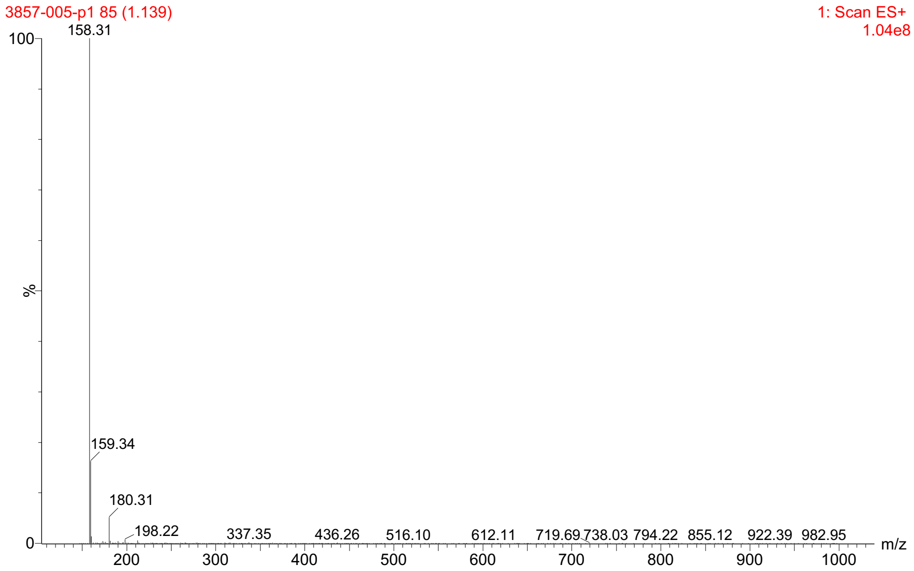

3. LCMS spectrum of Int-8 (batch# 3826-041-P2)

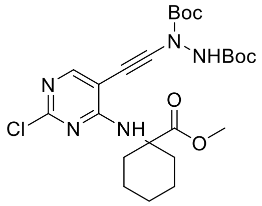

8

Molecular Weight: 524.02

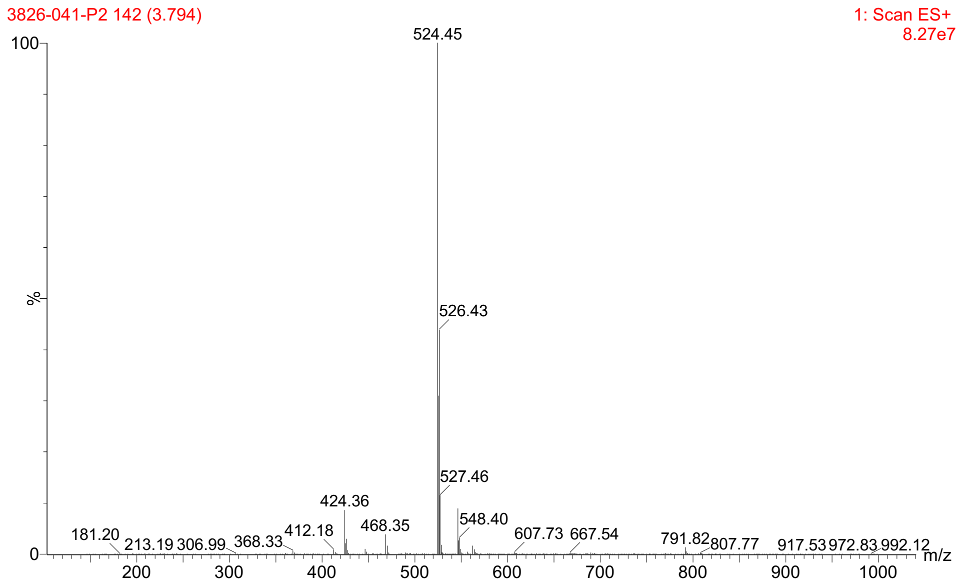

4. **LCMS spectrum of Int-11a (batch# 3797-040-P8)**

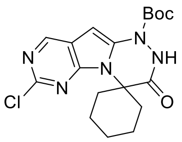

Intermediate 11a

Molecular Weight: 391.86

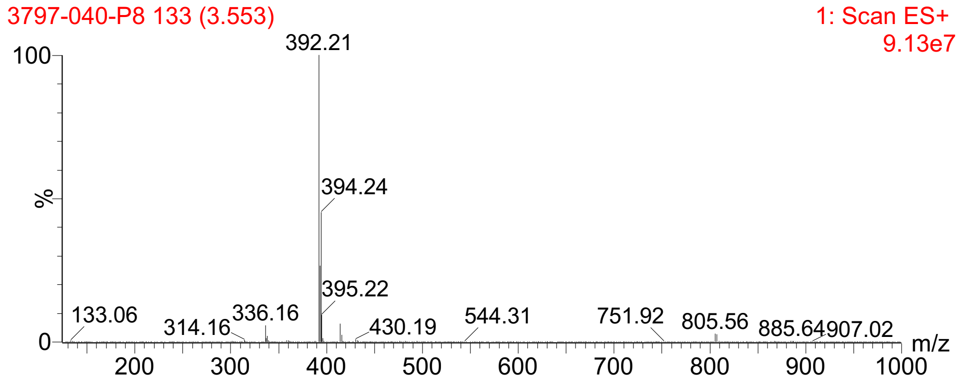

5. **LCMS spectrum of Int-12a (batch# 3797-046)**

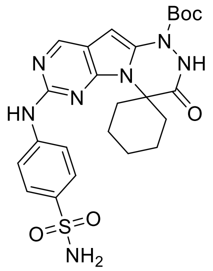

12a

Molecular Weight: 527.60

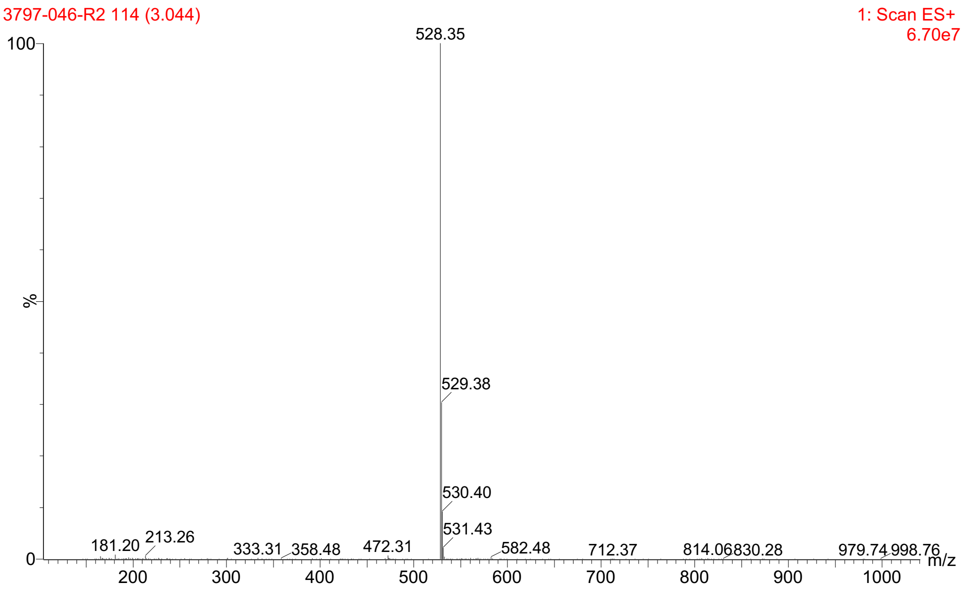

6. LCMS spectrum of INX-315 (batch# 3797-049-P2)

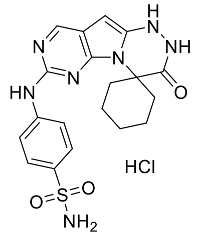

ARC-0315

Molecular Weight: 427.48

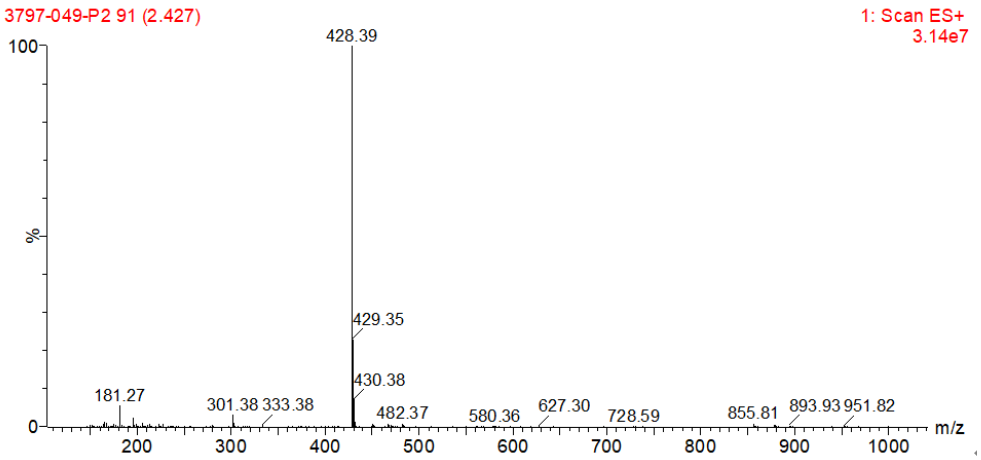

3797-049-P2 91 (2.427)
1: Scan ES+
3.14e7
100
%
0
428.39
429.35
430.38
181.27
301.38 333.38
482.37
580.36
627.30
728.59
855.81
893.93
951.82
200
300
400
500
600
700
800
900
1000
m/z
