# Supplementary material for: INX-315, a Selective CDK2 Inhibitor, Induces Cell Cycle Arrest and Senescence in Solid Tumors
Source: Cancer Discov. 2023 Dec 1;14(3):446–67. doi: 10.1158/2159-8290.CD-23-0954 (PMC10905675; doi:10.1158/2159-8290.CD-23-0954)
Supplement: Supplementary Table 1 — Experimental conditions for Nanosyn assays [file cd-23-0954_supplementary_table_1_suppst1.docx]

| Assay | Assay platform | Enzyme preparation (Vendor. catalog no. Lot no.) | [Enzyme], nmol/L | ATP Conc. (μmol/L) | Substrate concentration (μmol/L) | Incubation time (h) |
| --- | --- | --- | --- | --- | --- | --- |
| CDK1-Cyclin B | Caliper MSA | Millipore-14-450M-26383U | 0.08 | 40 | 1 | 2 |
| CDK2-Cyclin A | Caliper MSA | Millipore-14-448-23984 | 0.15 | 50 | 1 | 3 |
| CDK2-Cyclin E | Caliper MSA | Millipore-14-475-2166901-E | 0.15 | 100 | 1 | 3 |
| CDK3-Cyclin E | Caliper MSA | BPS-40103-110804-5 | 1 | 400 | 1 | 3 |
| CDK4-Cyclin D1 | Caliper MSA | ThermoFisher-PV4400-1754389T | 1 | 200 | 1 | 3 |
| CDK5-p25 | Caliper MSA | BPS-40105-130618-2 | 0.1 | 20 | 1 | 3 |
| CDK5-p35 | Caliper MSA | ThermoFisher-PV3000-25348A | 0.047 | 20 | 1 | 3 |
| CDK6-Cyclin D3 | Caliper MSA | Carna-04-107-15CBS-0744K | 2 | 300 | 1 | 3 |
| CDK7-Cyclin H | Caliper MSA | Carna-04-108-13CBS-0015 | 5 | 50 | 1 | 17 |
| CDK9-Cyclin T1 | Caliper MSA | Carna-04-110-14CBS-0084 | 3 | 10 | 1 | 17 |
