# Supplementary material for: INX-315, a Selective CDK2 Inhibitor, Induces Cell Cycle Arrest and Senescence in Solid Tumors
Source: Cancer Discov. 2023 Dec 1;14(3):446–67. doi: 10.1158/2159-8290.CD-23-0954 (PMC10905675; doi:10.1158/2159-8290.CD-23-0954)
Supplement: Supplementary Figures 1-14 — Supplementary Figure 1: Characterization of INX-315.Supplementary Figure 2: Effect of INX-315 and PF-07104091 on viability of cancer and non-malignant cell lines.Supplementary Figure 3: Effect of INX-315 on cell cycle in CCNE1-amplified cancer cell lines.Supplementary Figure 4: Further characterization of the effects of INX-315 in in vitro and in vivo models of CCNE1-amplified cancer.Supplementary Figure 5: Effects of INX-315 treatment on gene expression in models of CCNE1amplified ovarian carcinoma.Supplementary Figure 6: Characterization of CDK4/6 inhibitor-resistant cell lines.Supplementary Figure 7: Response of CDK4/6 inhibitor-resistant breast cancer cells to INX-315 and PF-07104091.Supplementary Figure 8: Effects of INX-315 and PF-07104091 on CDK4/6 inhibitor-resistant breast cancer cells.Supplementary Figure 9: Impact of INX-315 +/- continued CDK4/6 inhibition in CDK4/6 inhibitorresistant breast cancer cells.Supplementary Figure 10: Characterization of a new mouse model of acquired CDK4/6 inhibitor resistance.Supplementary Figure 11: Effect of INX-315 on expression of senescence-related genes in CDK4/6 inhibitor-resistant breast cancer cells.Supplementary Figure 12: Epigenomic and transcriptomic features of INX-315 induced senescence in CDK4/6 inhibitor-resistant breast cancer.Supplementary Figure 13: Impact of INX-315 treatment of CDK4/6 inhibitor resistant breast cancer on expression of apoptosis and differentiation-related genes.Supplementary Figure 14: Impact of co-inhibition of CDK2 and CDK4/6 on cell cycle and development of CDK4/6 inhibitor resistance in breast cancer cells. [file cd-23-0954_supplementary_figures_1-14_suppsf1-sf14.pdf]

Supplementary Figure 1

A

Table 1: INX-315 Physiochemical Properties	
Molecular Weight	427.48
Molecular Formula	$C_{19}H_{21}N_7O_3S$
Appearance	White to tan powder
Form	Crystalline
Water Solubility	<50 µg/mL
Polyethylene glycol (PEG) 400 Solubility	>100 mg/mL
pKa(s)	10.42 (acidic); 5.08 (basic)
cLogP	2.381

B

Table 2: Half-lifes

Compound	Species	Half-life[a] (min)
Trilaciclib	Human	>60 (66)
	Rat	>60 (73)
	Mouse	28
	Dog	5
Compound B	Human	<10
	Rat	<10
	Mouse	<10
	Dog	<10
Compound C	Human	15.3
	Rat	<10
	Mouse	<10
	Dog	23.4
INX-315	Human	>60 (107)
	Rat	>60 (66.6)
	Mouse	26.4
	Dog	>60 (80.6)

[a] When the calculated half-life was longer than the duration of the experiment, the half-life was expressed as > the longest incubation time. Then, if the calculated half-life was <2x the duration of the experiment, the calculated half-life was listed in parenthesis.

C

	INX-315	PF-07104091
	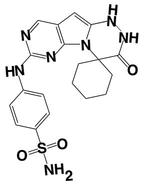	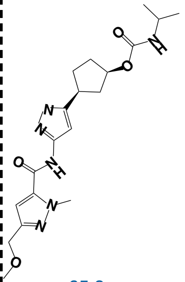
IC_{50}(nM):		
CDK1/B1	30 (8.3)	65.8
CDK2/cyclin E1	0.6 (0.2)	2.4
CDK2/cyclin A2	2.5 (0.7)	7.4
CDK3/cyclin E1	15.1 (0)	ND
CDK4/cyclin D1	126 (29.2)	>1000
CDK5	23.1 (0)	ND
CDK5/p25	21.4 (0)	ND
CDK6/cyclin D3	349 (88.4)	>1000
CDK7	>10000 (0)	ND
CDK9/cyclin T1	62 (26.0)	363

D

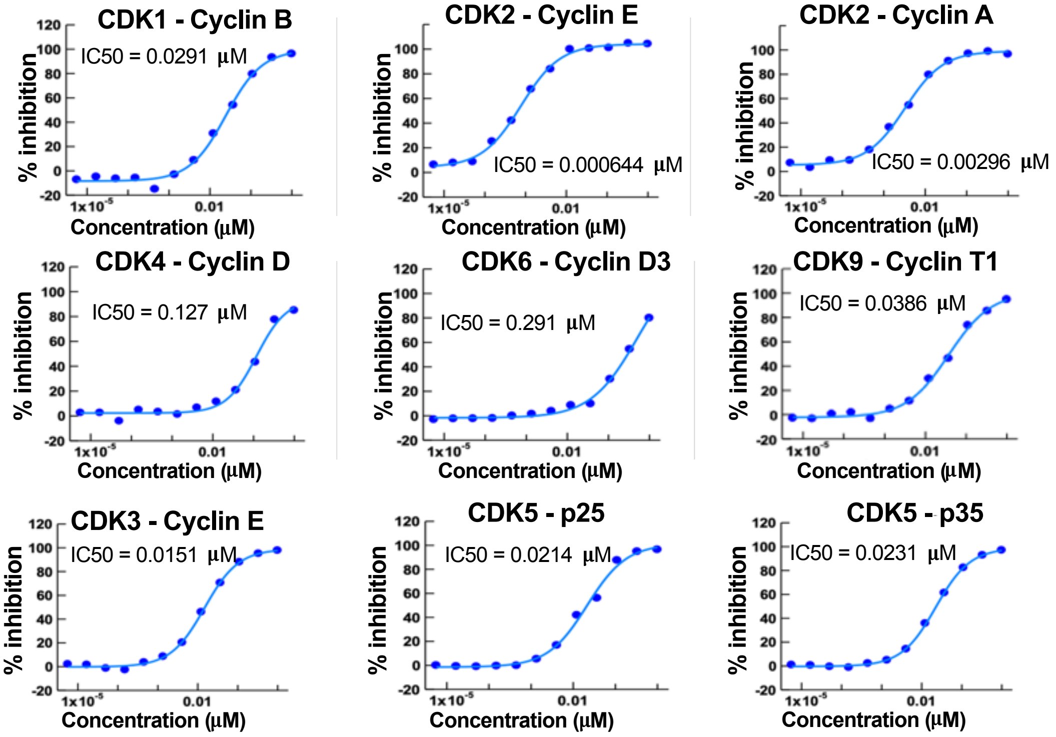

E

PF-07104091 Intracellular IC_{50}

	CDK2/E1	CDK2/A1	CDK1/B1	CDK9/T1
IC_{50}(nM)	32	342	1180	>10000
Fold vs CDK2/E1	1	11	37	>313

F

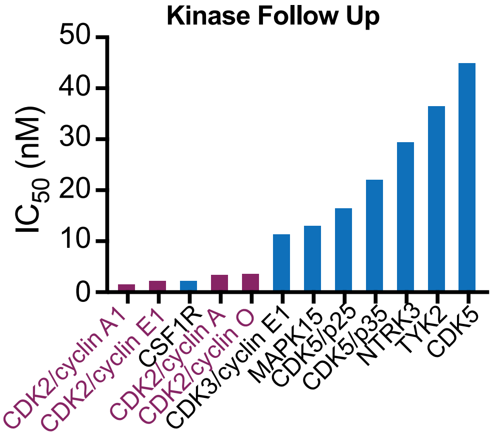

Supplementary Figure 1: Characterization of INX-315. **(A)** Physico-chemical properties of INX-315. **(B)** Half-lives of various compounds using a species-specific microsomal stability assay (n=1). When the calculated half-life was longer than the duration of the experiment, the half-life is expressed as "> the longest incubation time". Then, if calculated half-life was <2x the duration of the experiment, the calculated half-life is listed in parentheses. **(C)** Table indicates biochemical IC_{50}s (+/- SEM) to cyclin/CDK pairings shown using the Nanosyn biochemical assay for INX-315 and PF-07104091. (INX-315: if SEM is 0, n =1, otherwise n = 6; PF-07104091 n=1 for all complexes). **(D)** Representative dose response curves demonstrating INX-315 inhibition of cyclin/CDK complexes using the Nanosyn assay, as in (C). **(E)** NanoBRET assay quantifying PF-07104091's intracellular displacement of tracer from the ATP-pocket of the cyclin/CDK pairings shown (2 technical replicates, 1 biological replicate). **(F)** Biochemical inhibitory IC_{50}s for INX-315 against kinases shown, based on 10-point dose-response curve (n=1).

Supplementary Figure 2

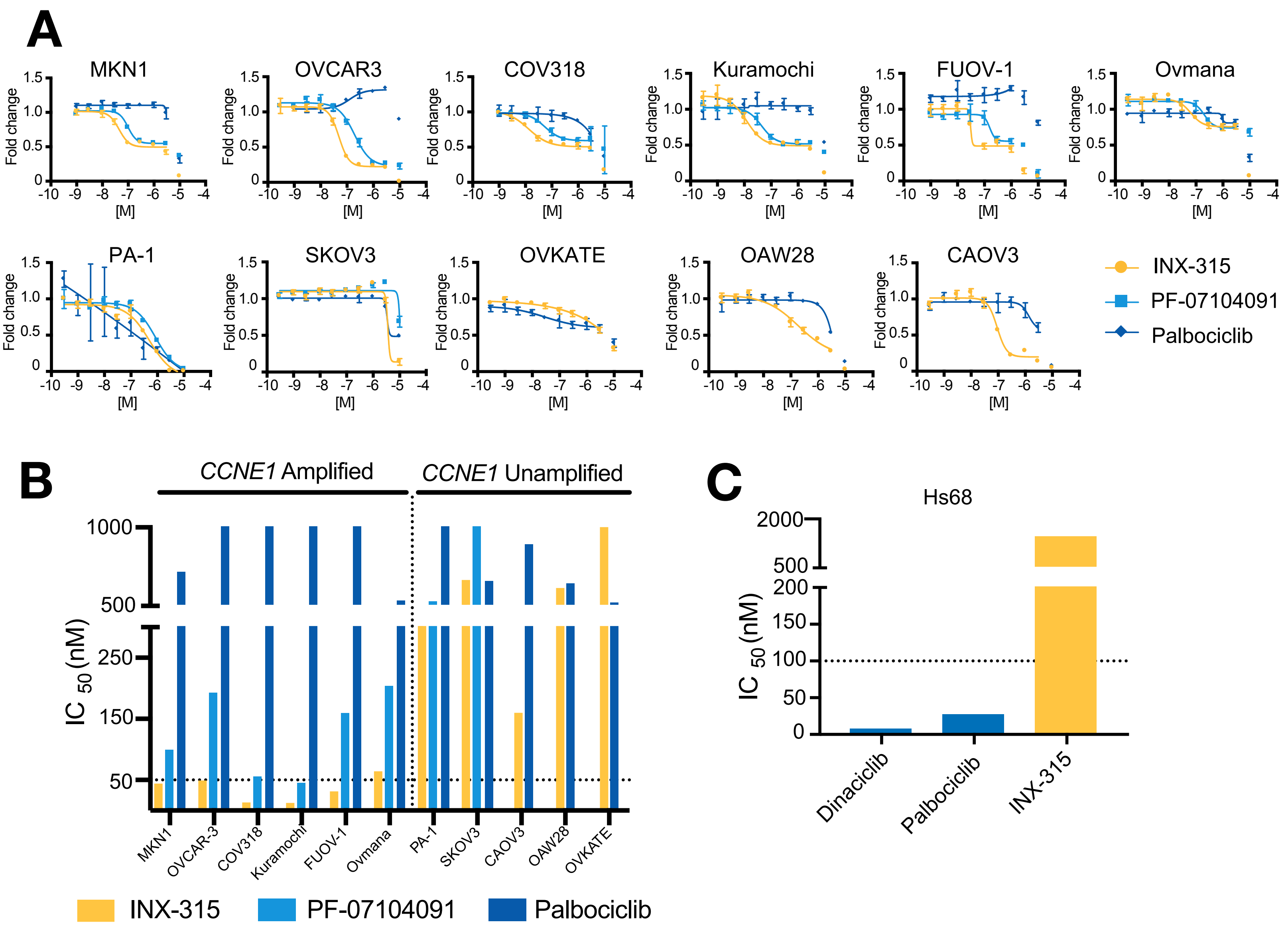

Supplementary Figure 2: Effect of INX-315 and PF-07104091 on viability of cancer and non-malignant cell lines. (A) Cell Titer Glo viability assay dose response curves for cancer cell lines treated with drugs indicated, used to calculate data presented in Fig. 2A. **(B)** Comparison of IC50 values for INX-315 and PF-07104091 for the cell lines shown, calculated from (A). Cells were treated for 6 days (3 technical replicates). **(C)** IC50s for dinaciclib, palbociclib and INX-315 for Hs68 cells calculated from Cell Titer Glo viability assay (3 technical replicates).

Supplementary Figure 3

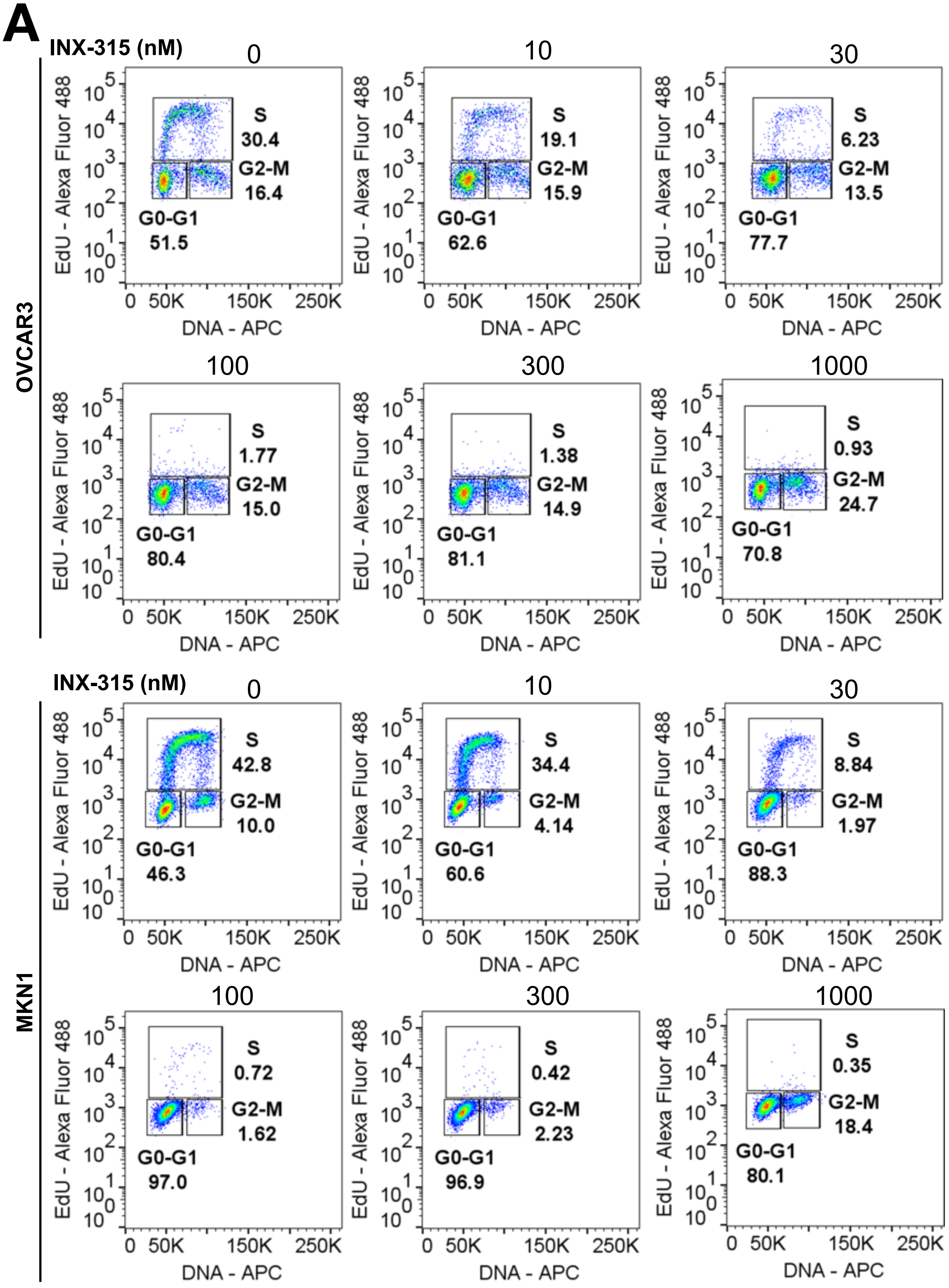

Supplementary Figure 3: Effect of INX-315 on cell cycle in *CCNE1*-amplified cancer cell lines. (A) Representative flow cytometry plots profiling cell cycle phases (by measuring DNA content and EdU incorporation) in OVCAR3 and MKN1 cells treated with INX-315, as in Fig. 2B. Representative of 3 replicates.

Supplementary Figure 4

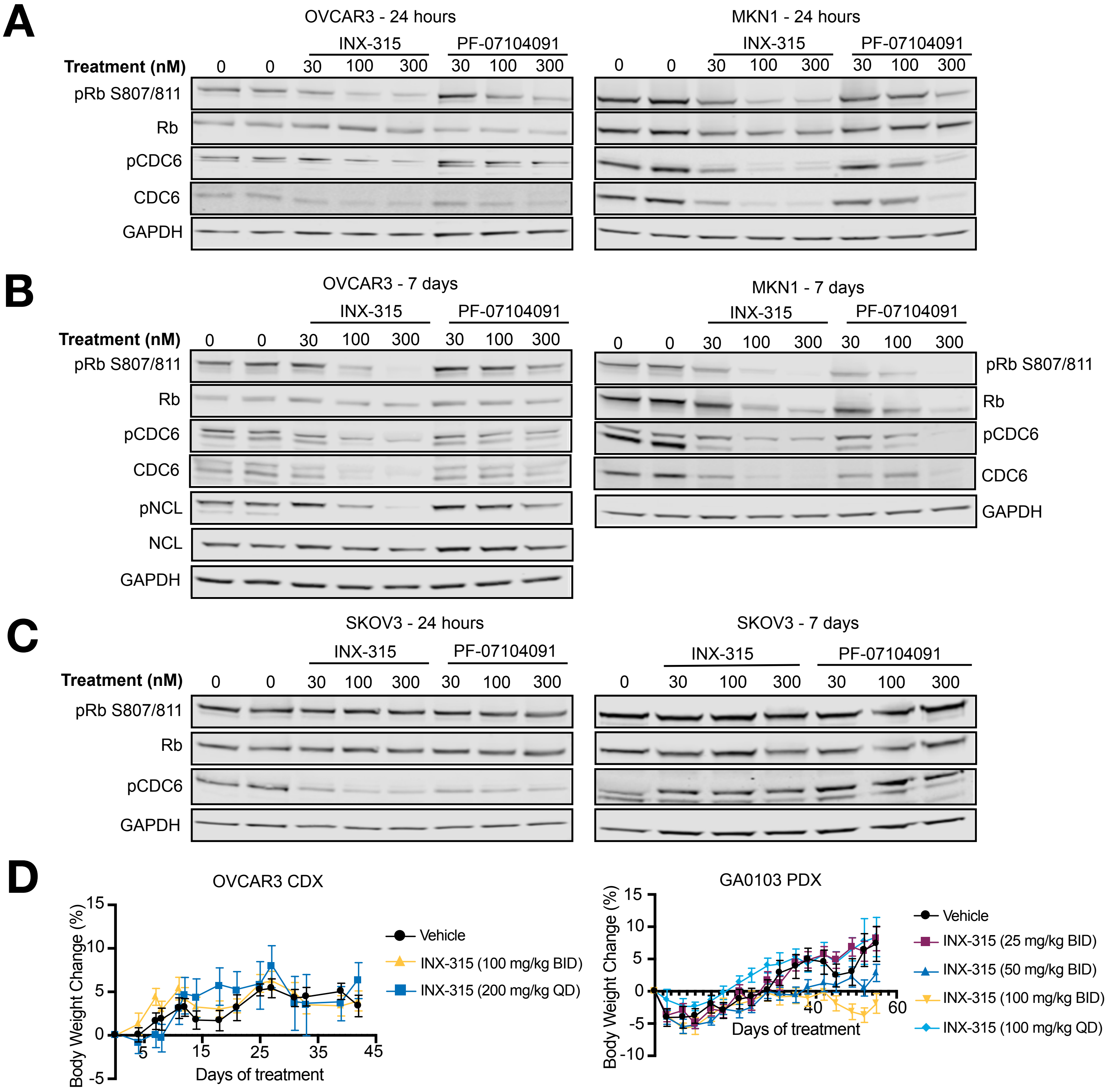

Supplementary Figure 4: Further characterization of the effects of INX-315 in in vitro and in vivo models of *CCNE1*-amplified cancer. Western blots for phosphorylated and total proteins shown in OVCAR3 and MKN1 cells treated with INX-315 or PF-017104091 for 24 hours **(A)** or 7 days **(B),** n =1. **(C)** Western blots for phosphorylated and total proteins in SKOV3 cells treated with INX-315 or PF-017104091 for 24 hours or 7 days, n=1. **(D)**Weight of tumor-bearing mice during treatment with INX-315 at doses shown or control vehicle. Tumor growth curves from this experiment are in Figs. 2D and 2E (error bars represent SEM).

Supplementary Figure 5

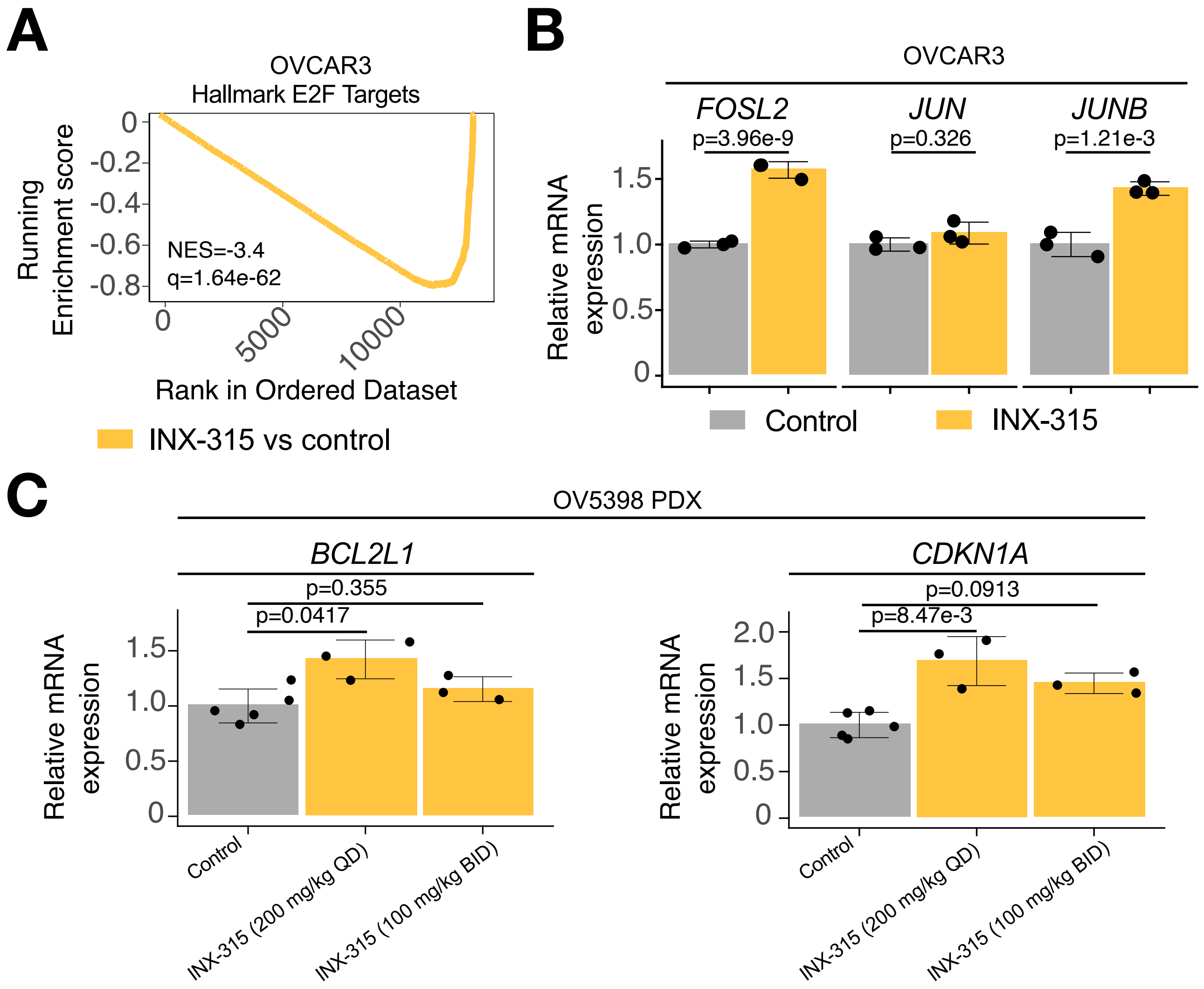

Supplementary Figure 5: Effects of INX-315 treatment on gene expression in models of *CCNE1*-amplified ovarian carcinoma. (A) GSEA plots derived from RNA-seq performed on OVCAR3 cells treated with 300 nM INX-315 for 7 days. NES (normalized enrichment score) and q value (false discovery rate) were calculated as described in Methods. 3 technical replicates per condition. **(B)** Relative expression of *FOSL2, JUN,* and *JUNB* genes in OVCAR3 cells treated with control or 300 nM INX-315 or 7 days, derived from RNA-seq in (A). **(C)** Relative expression of *BCL2L1* and *CDKN1A* genes in OV5398 PDX tumor tissue treated with control or INX-315 at doses shown, derived from RNA-seq. Tumor tissue was collected at experimental endpoint from experiment in Fig. 2E (all p-values from DESeq2, adjusted for multiple comparisons, error bars represent SD).

Supplementary Figure 6

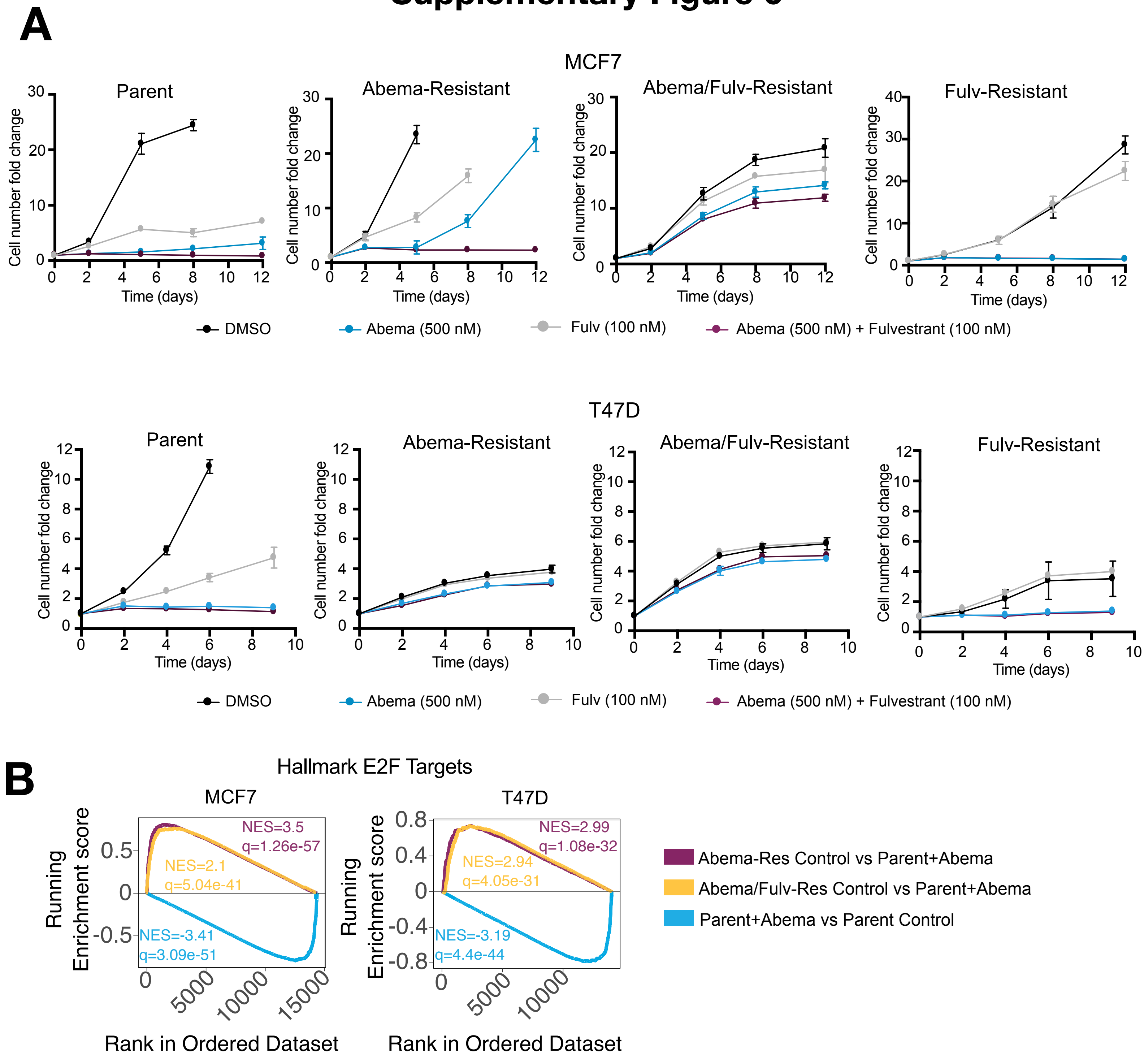

Supplementary Figure 6: Characterization of CDK4/6 inhibitor-resistant cell lines. (A) Growth of MCF7 and T47D cells over time demonstrating drug-sensitive and drug-resistant phenotypes (Error bars represent SD). **(B)** GSEA plots derived from RNA-seq performed on MCF7 and T47D cells. Parent cells growing in control vehicle or 500 nM abemaciclib; abemaciclib-resistant cells growing in 500 nM abemaciclib; abemaciclib/fulvestrant-resistant cells growing in 500 nM abemaciclib + 100 nM fulvestrant. NES (normalized enrichment score) and q value (false discovery rate) were calculated as described in Methods.

Supplementary Figure 7

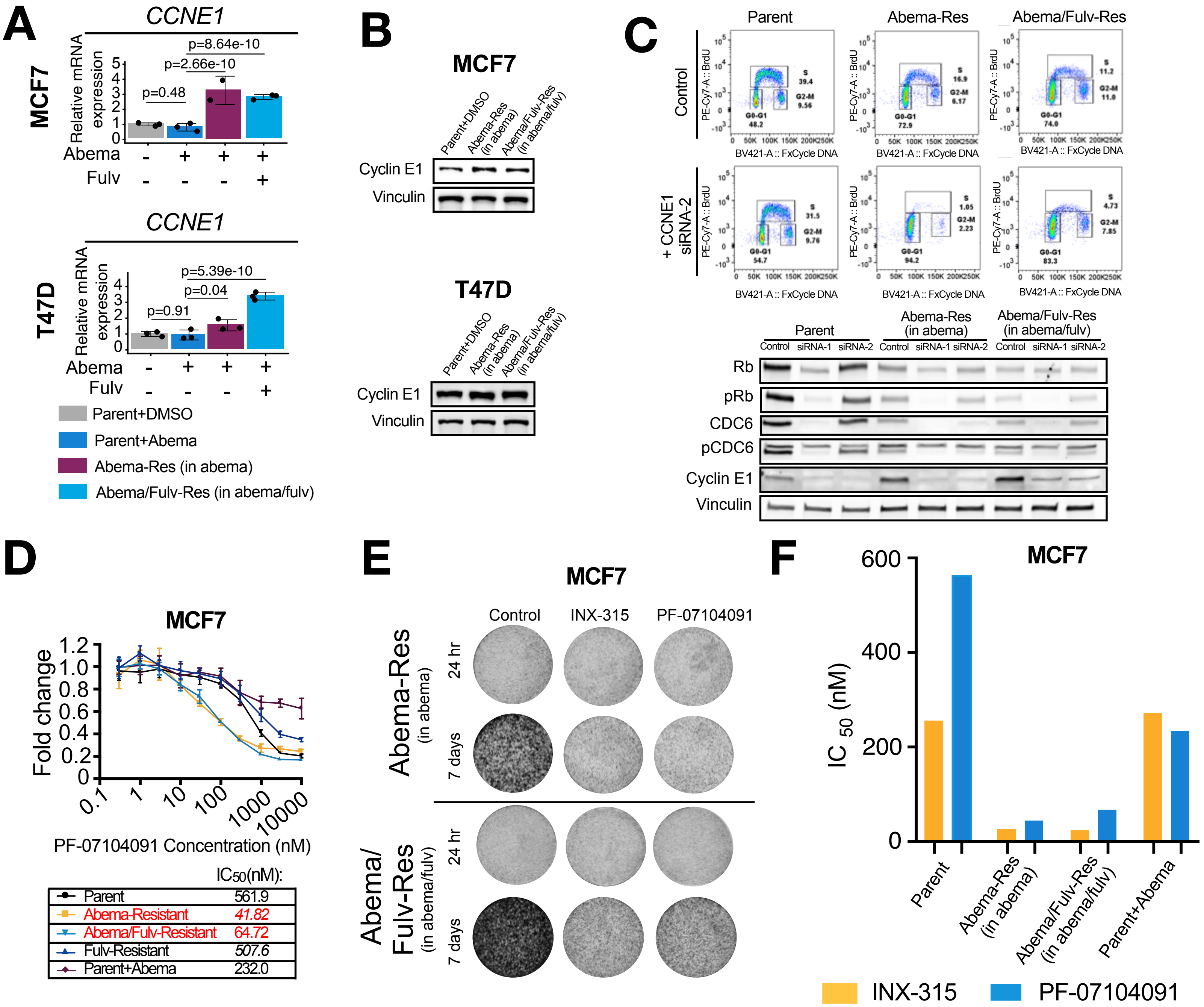

Supplementary Figure 7: Response of CDK4/6 inhibitor-resistant breast cancer cells to INX-315 and PF-07104091. (A) Relative *CCNE1* gene expression in parental and drug-resistant MCF7 and T47D cells, derived from RNA-seq. **(B)** Western blots showing cyclin E1 protein level in parental and CDK4/6 inhibitor-resistant cell lines. **(C)** Impact of *CCNE1* knockdown with siRNA on cell cycle kinetics in abemaciclib- and abemaciclib/fulvestrant-resistant MCF7 cells. Western blot demonstrates degree of *CCNE1* knockdown and confirm reduced phosphorylation of CDK2 substrates. Representative flow cytometry plots cells depict cell cycle phases determined by measuring DNA content and BrdU incorporation); n=3 technical replicates. **(D)** Dose response curves for MCF7 cells treated with PF-07104091 for 7 days. Parent: parent cells in DMSO; Abema-resistant: abemaciclib-resistant cells growing in 500 nM abemaciclib; Abema/Fulv-resistant: resistant to abemaciclib/ fulvestrant growing in 500 nM abemaciclib plus 100 nM fulvestrant; Fulv-resistant: fulvestrant resistant growing in 100 nM fulvestrant; Parent + abema: parental cells treated with 500 nM abemaciclib and PF-07104091 concurrently; 6 technical replicates per condition. **(E)** Representative images from a clonogenic assay in which CDK4/6 inhibitor-resistant MCF7 cells were treated with control vehicle, abemaciclib (500 nM), fulvestrant (100 nM) PF-07104091 (300 nM), INX-315 (300 nM) or various combinations as shown; 2 biological replicates with 2 technical replicates per condition. **(F)** Bar graph showing IC50 for INX-315 and PF-07104091 for parental and drug-resistant MCF7 cells (NB. INX-315 data obtained from a separate experiment directly comparing to PF-07104091, distinct from experiment in Fig. 4A).

Supplementary Figure 8

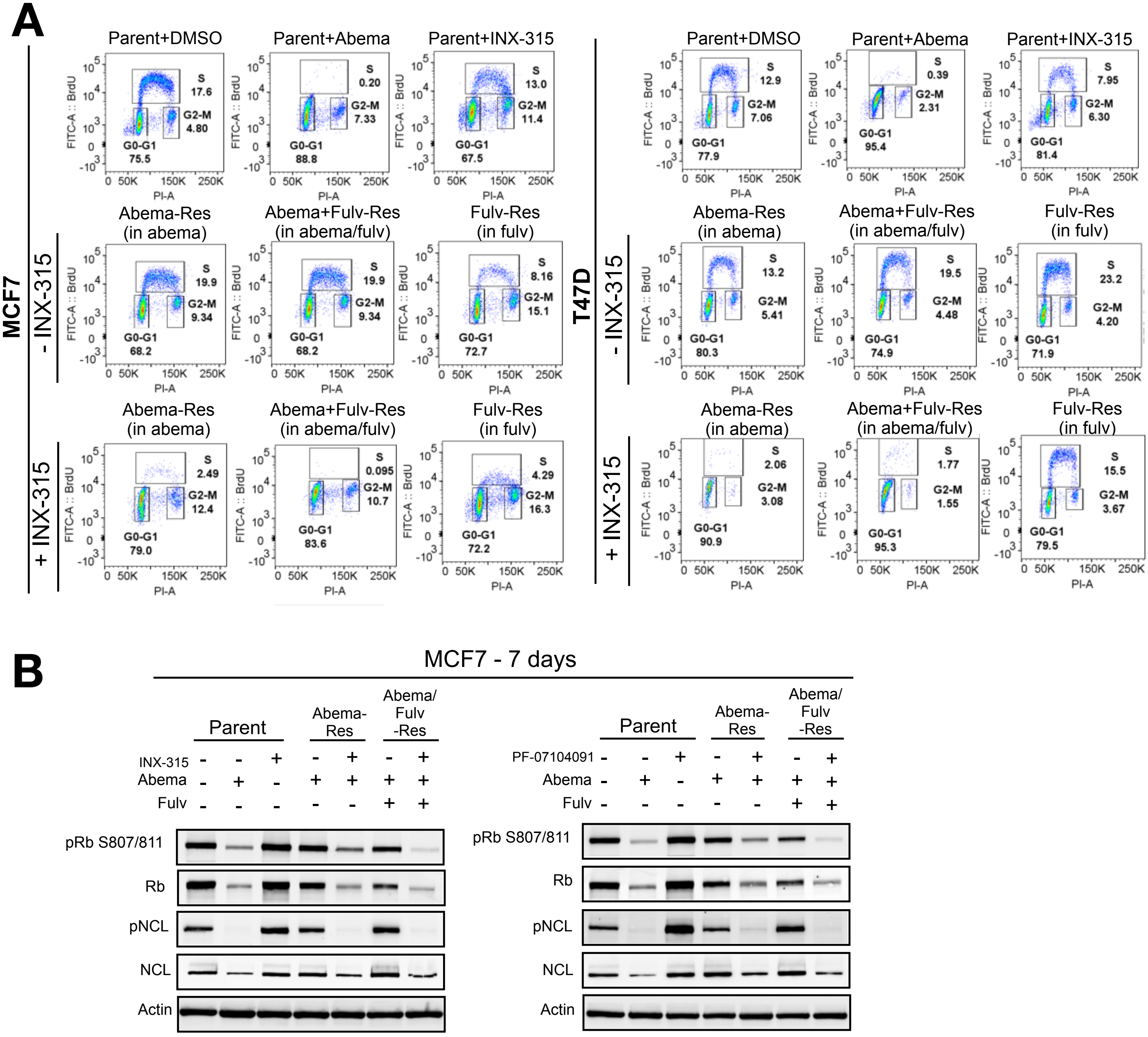

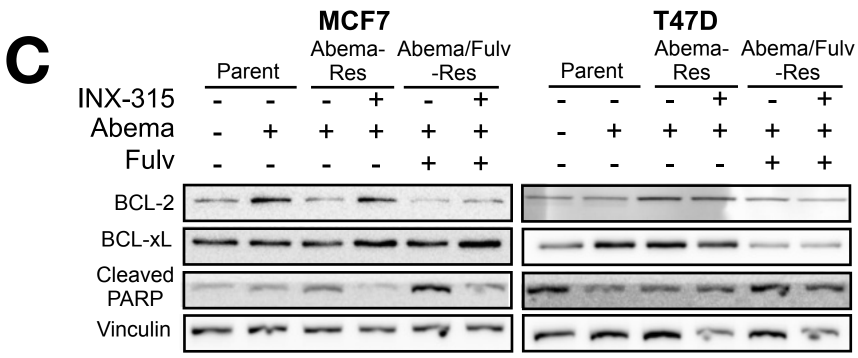

Supplementary Figure 8: Effects of INX-315 and PF-07104091 on CDK4/6 inhibitor-resistant breast cancer cells. (A) Representative flow cytometry plots profiling cell cycle phases (by measuring DNA content and BrdU incorporation) in MCF7 and T47D cells, treated for 7 days with drugs as in Fig. 4B. NB: drug resistant cells were cultured in drugs they were resistant to +/- INX-315. Representative of 2 replicates. **(B)** Western blots showing phosphorylated and total nucleolin levels in parental and drug-resistant MCF7 cells treated with drugs shown for 7 days (500 nM abemaciclib, 100 nM fulvestrant, 300 nM PF-07104091, 300 nM INX-315. n = 1 **(C)** Western blots for Bcl-2, Bcl-xL, and Cleaved PARP in MCF7 and T47D cells treated for 7 days as in Fig. 4B (n =1).

Supplementary Figure 9

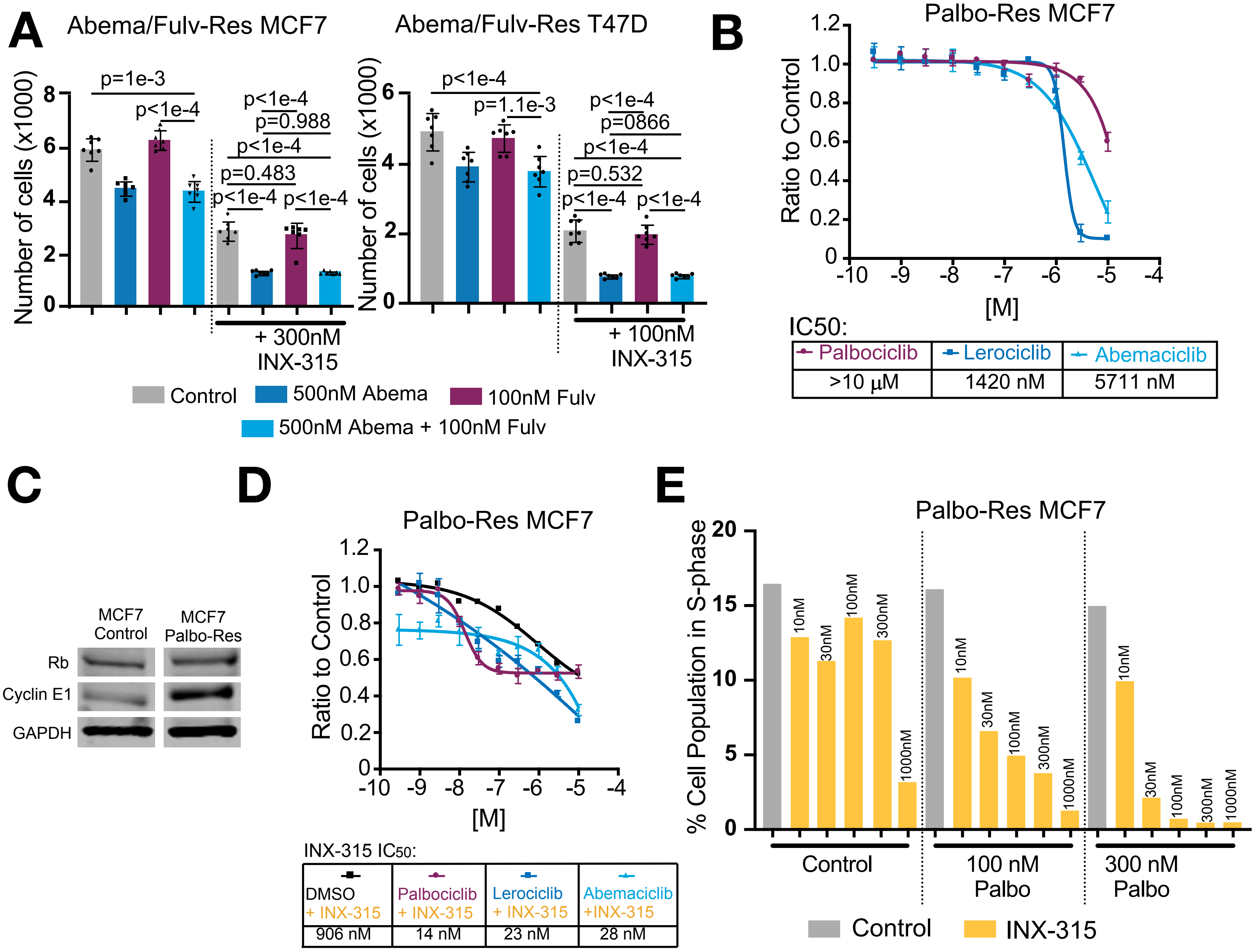

Supplementary Figure 9: Impact of INX-315 +/- continued CDK4/6 inhibition in CDK4/6 inhibitor-resistant breast cancer cells. (A) Abemaciclib/fulvestrant-resistant MCF7 and T47D cells were cultured in the drugs shown for 2 doubling times (doubling time of the cells growing in abemaciclib/fulvestrant), followed by determination of cell number (6 replicates, error bars represent SD, p values calculated using unpaired t-tests). **(B)** MCF7 cells cultured to resistance in palbociclib were treated with different CDK4/6 inhibitors. IC50s are shown, calculated using results of Cell Titer Glo assay (error bars represent SEM; 3 technical replicates). **(C)** Western blot showing levels of cyclin E1 in parental and palbociclib-resistant MCF7 cells. n =1 **(D)** Palbociclib-resistant MCF7 cells were cultured in drugs shown (CDK4/6 inhibitors at 1 μM; INX-315 on a dose-response curve). IC50 for INX-315 is shown for each condition (error bars represent SEM; 3 technical replicates). **(E)** Palbociclib-resistant MCF7 cells were treated with drugs as shown, and cell cycle phase quantification was performed. Graph shows fraction of cells in S phase (n =1).

Supplementary Figure 10

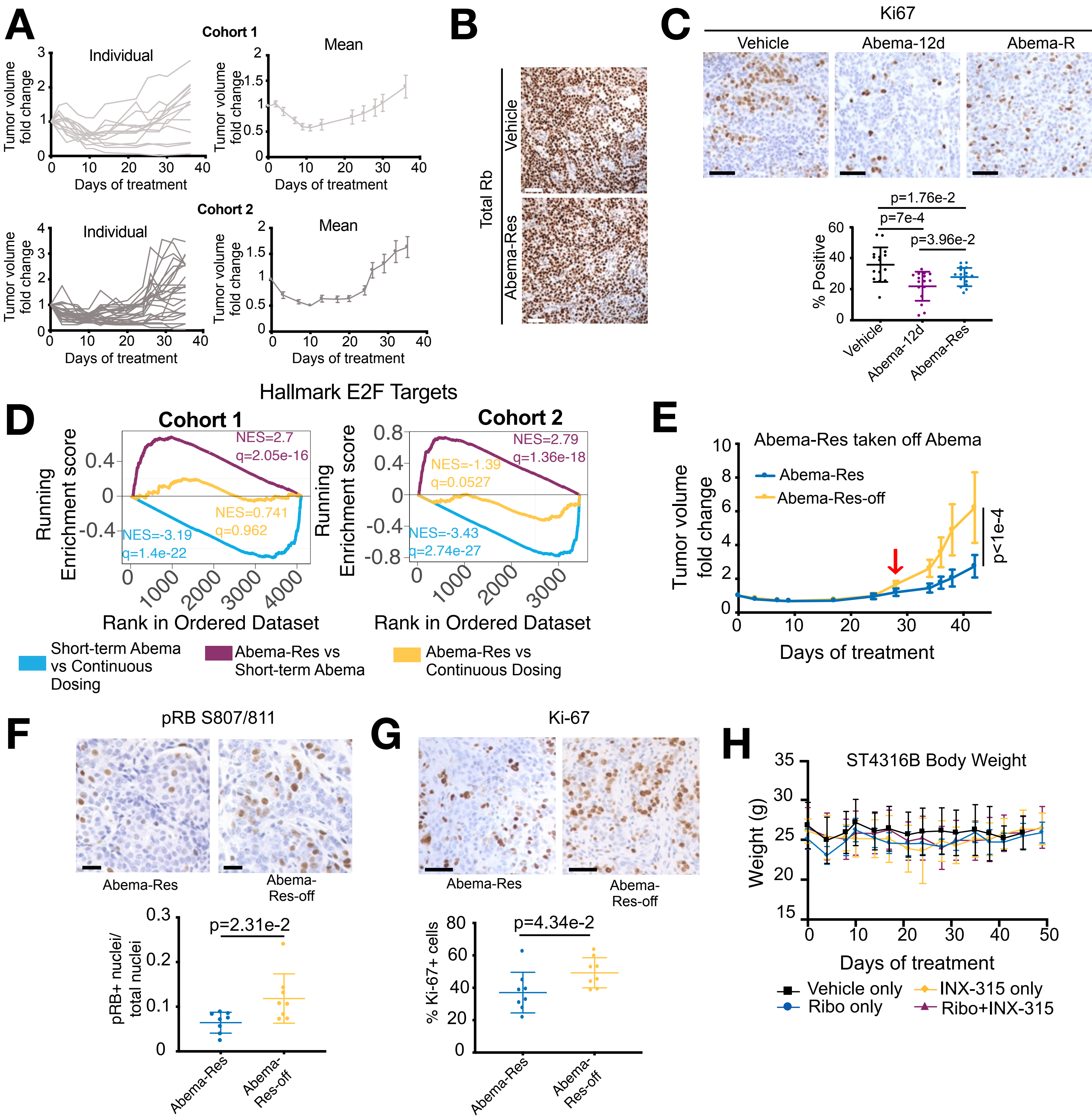

Supplementary Figure 10: Characterization of a new mouse model of acquired CDK4/6 inhibitor resistance. **(A)** Individual (left) and mean (right) tumor growth curves for representative cohorts of tumor bearing *MMTV-rtTA/tetO-HER2* mice treated with abemaciclib (cohort 1: n=13; cohort 2: n=24). **(B)** Immunohistochemistry for Rb in abemaciclib-naïve and abemaciclib-resistant *MMTV-rtTA/tetO-HER2* tumors (scale bar = 50 µm). **(C)** Top: representative images of Ki67 immunohistochemistry in *MMTV-rtTA/tetO-HER2* tumors treated with control vehicle for 36 days (vehicle, n=15), control vehicle for 24 days followed by 12 days of abemaciclib ("responding", n=16), or abemaciclib for 36 days, at which time resistance was observed ("resistant", n=16) (scale bar = 50 µm). Bottom: quantification of Ki67 staining. **(D)** GSEA plots derived from RNA-seq performed on tumors as in (C). NES (normalized enrichment score) and q value (false discovery rate) were calculated as described in Methods. **(E)** Growth curves of *MMTV-rtTA/tetO-HER2* tumors treated with abemaciclib until acquired resistance (red arrow), followed either by continued abemaciclib (Abema-Res, n=23) or vehicle (Abema-Res-off, n=23). **(F)** and **(G)** Representative images and quantification of immunohistochemistry for p-Rb (scale bar = 25 µm) and Ki67 (scale bar = 50 µm) from tumors as in (E). **(H)** Weight of PDX ST4316B tumor-bearing mice during treatment with agents shown. Tumor growth curves from this experiment are in Fig. 4G. n = 8 [for (C), (F), and (G) error bars are SD and p values were calculated using unpaired t-tests; for (A), (E), and (H) error bars are SEM and are p value is by two-way ANOVA].

Supplementary Figure 11

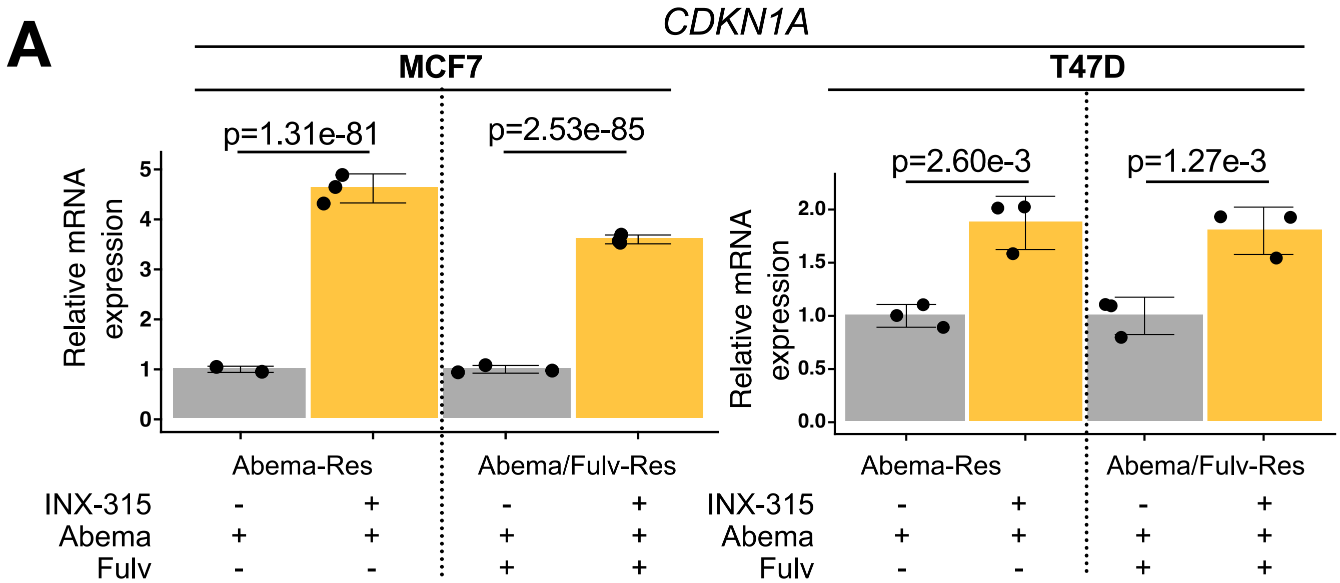

Supplementary Figure 11: Effect of INX-315 on expression of senescence-related genes in CDK4/6 inhibitor-resistant breast cancer cells. (A) Abemaciclib-resistant and abemaciclib/fulvestrant-resistant cells were treated with drugs shown for 7 days. Graphs show relative expression of *CDKN1A* as derived from RNA-seq (all p values from DESeq2, adjusted for multiple comparisons; error bars represent SD; 3 technical replicates).

Supplementary Figure 12

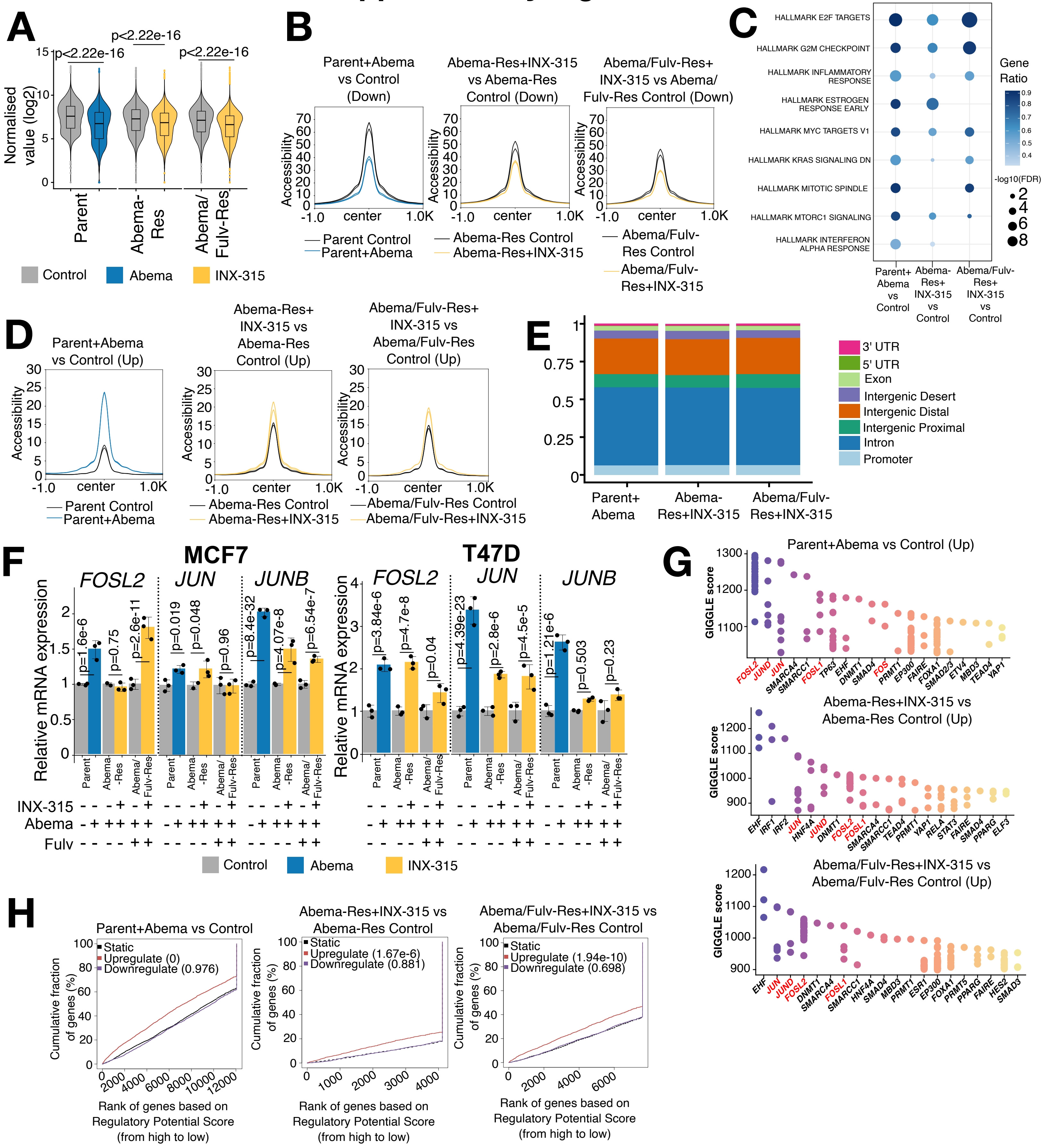

Supplementary Figure 12: Epigenomic and transcriptomic features of INX-315 induced senescence in CDK4/6 inhibitor-resistant breast cancer. (A) MCF7 cells were treated for 7 days as follows: parent cells – DMSO or 500 nM abemaciclib; abemaciclib-resistant cells – 500 nM abemaciclib or 500 nM abemaciclib + INX-300 nM 315; abemaciclib/fulvestrant-resistant cells – 500 nM abemaciclib /100 nM fulvestrant vs 500 nM abemaciclib /100 nM fulvestrant + 300 nM INX-315. Violin plot shows log2-trasformed normalized ATAC-seq counts for regions with decreased chromatin accessibility upon abemaciclib treatment of parental cells (p values calculated using unpaired t-tests; ATAC-seq done with 2 technical replicates). **(B)** Visualization of ATAC-seq signal at regions with decreased chromatin accessibility after abemaciclib treatment of parental cells. **(C)** Bubble plots showing gene-set enrichment analysis results from ChIP-Enrich on regions with decreased chromatin accessibility after drug treatment as in (A). **(D)** Visualization of ATAC-seq signal at regions with increased chromatin accessibility after abemaciclib treatment of parent cells. **(E)** Genomic distribution of regions showing significantly increased chromatin accessibility by ATAC-seq in cells treated as in (A). **(F)** Relative expression of *FOSL2, JUN,* and *JUNB* in cells treated as in (A), derived from RNA-seq data (all p values from DESeq2, adjusted for multiple comparisons; error bars represent SD; 3 technical replicates). **(G)** Similarity (GIGGLE) scores between regions of increased ATAC-seq signal genome-wide in MCF7 cells treated as in (A) and GEO-archived datasets of ChIP-seq for transcription factors (using 'Cistrome Toolkit'). Top-ranked factors are shown and AP-1 factors are labelled red. **(H)** Binding expression and target analysis (BETA) of MCF7 cells treated as in (A). Graphs show association of ATAC-seq up-peaks containing Jun motifs and expression of nearby genes on RNA-seq. p values (BETA statistic) denote significance of association relative to background and demonstrate that regions of increased chromatin accessibility containing Jun motifs are strongly associated with upregulation of nearby genes.

Supplementary Figure 13

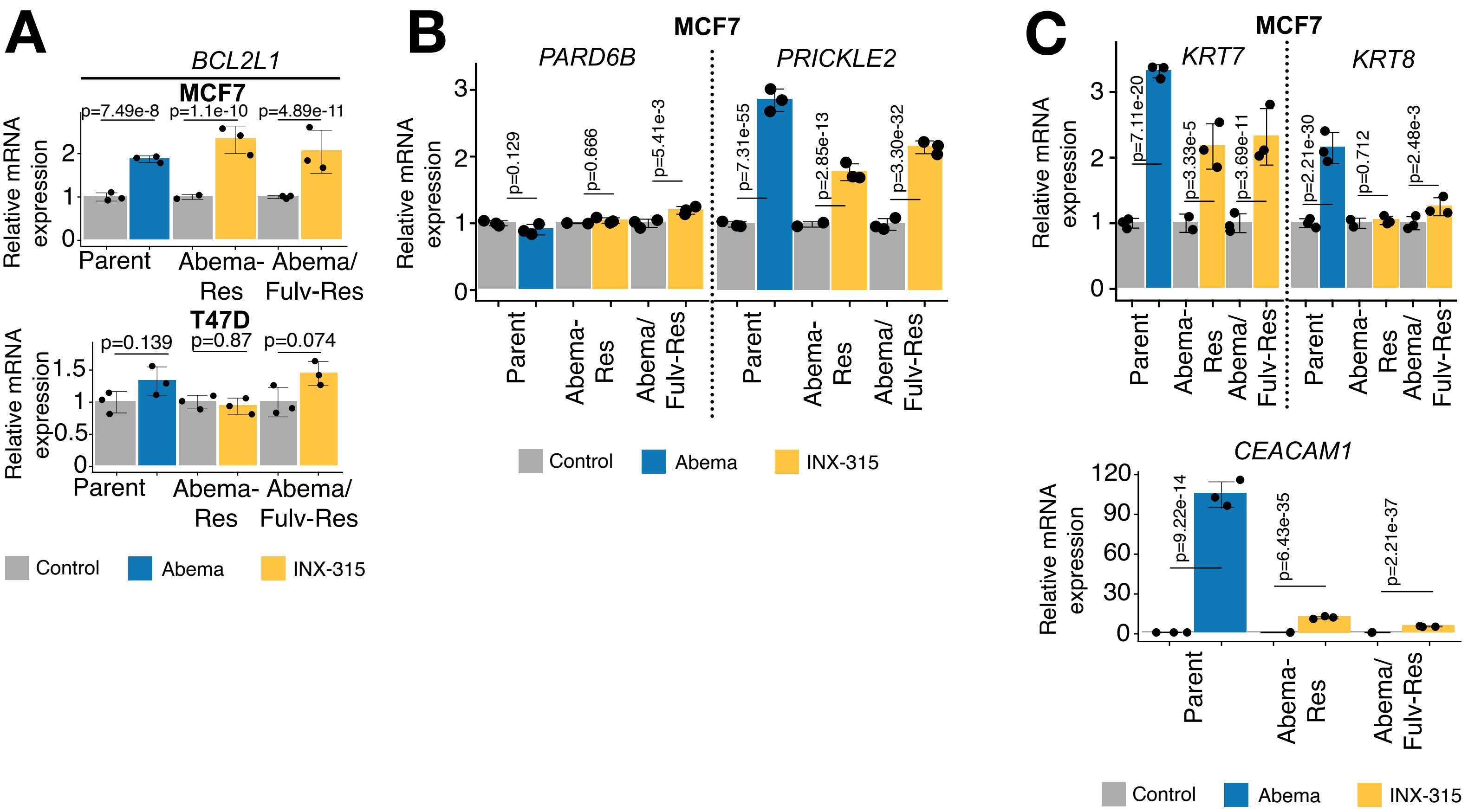

Supplementary Figure 13: Impact of INX-315 treatment of CDK4/6 inhibitor resistant breast cancer on expression of apoptosis and differentiation-related genes. MCF7 and T47D cells were treated for 7 days as follows: parent cells – DMSO or 500 nM abemaciclib; abemaciclib-resistant cells – 500 nM abemaciclib or 500 nM abemaciclib + INX-315 (300 nM for MCF7; 100 nM for T47D); abemaciclib/fulvestrant-resistant cells – 500 nM abemaciclib /100 nM fulvestrant vs 500 nM abemaciclib /100 nM fulvestrant + INX-315 (300 nM for MCF7; 100 nM for T47D). Graphs show relative expression of genes associated with apoptosis **(A)**, gland morphogenesis **(B),** and luminal differentiation **(C),** as derived from RNA-seq) (all p values from DESeq2, adjusted for multiple comparisons; error bars represent SD; 3 technical replicates).

Supplementary Figure 14

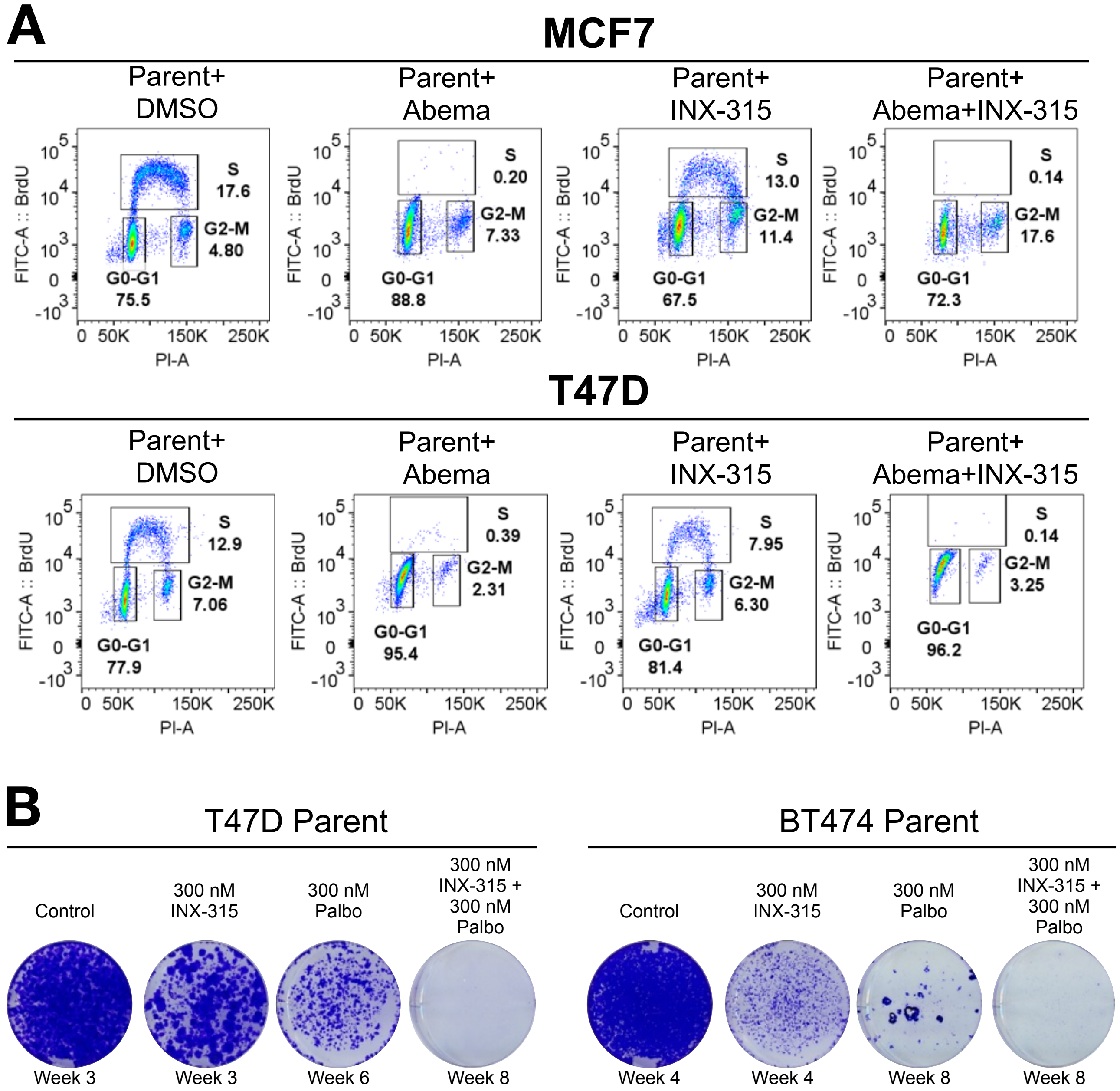

Supplementary Figure 14: Impact of co-inhibition of CDK2 and CDK4/6 on cell cycle and development of CDK4/6 inhibitor resistance in breast cancer cells. (A) Representative flow cytometry plots profiling cell cycle phases (by measuring DNA content and BrdU incorporation) in MCF7 and T47D cells treated with control, 500 nM abemaciclib, INX-315 (300 nM for MCF7, 100 nM for T47D), or the combination for 7 days. Representative of 2 replicates) **(B)** Representative images from a clonogenic assay in which T47D and BT474 cells were treated with control vehicle, palbociclib (300 nM), INX-315 (300 nM), or the combination. Representative of 3 technical replicates and 2 biological replicates.
